# Supplementary material for: Deep immune profiling delineates hallmarks of disease heterogeneity in extrapulmonary tuberculosis
Source: Nat Commun. 2025 Nov 10;16:9662. doi: 10.1038/s41467-025-65561-x (PMC12603278; doi:10.1038/s41467-025-65561-x)
Supplement: Supplementary file 1 — Supplementary Information [file 41467_2025_65561_MOESM1_ESM.pdf]

Supplementary Information

Deep immune profiling delineates hallmarks of disease heterogeneity in extrapulmonary tuberculosis

Sebastian J. Theobald[1,2,3,*], Kilian Dahm[4,5,6,*], Dinah Lange[1,2,3,*], Jannis B. Spintge[4], Sandra Winter[1,2], Angela Klingmüller[1,2,3], Lisa Holsten[4,5], Alexander Simonis[1,2,3], Elena De Domenico[4,7], Henning Walczak[8,9,10], Martina van Uelft[4,5], Joachim L. Schultze[4,5,7], Marc D. Beyer[4,7,11], Thomas Ulas[4,5,7,*,#], Isabelle Suárez[1,2,3,*], Jan Rybniker[1,2,3,*,#]

1. Department I of Internal Medicine, Medical Faculty and University Hospital Cologne, University of Cologne, 50937 Cologne, Germany
2. Center for Molecular Medicine Cologne (CMMC), Medical Faculty and University Hospital Cologne, University of Cologne, 50937 Cologne, Germany
3. German Center for Infection Research (DZIF), Bonn-Cologne, Germany
4. Systems Medicine, German Center for Neurodegenerative Diseases (DZNE), Bonn, Germany
5. Genomics and Immunoregulation, Life & Medical Sciences (LIMES) Institute, University of Bonn, Bonn, Germany
6. Translational Pediatrics, Department of Pediatrics, University Hospital Wuerzburg, 97080, Würzburg, Bavaria, Germany.
7. Platform for Single Cell Genomics and Epigenomics at the German Center for Neurodegenerative Diseases, the University of Bonn and West German Genome Center (WGGC), Bonn, Germany
8. Centre for Cell Death, Cancer, and Inflammation (CCCI), UCL Cancer Institute, University College London, London, UK.
9. Institute of Biochemistry I, Medical Faculty, University of Cologne, Cologne, Germany.
10. CECAD Research Centre, University of Cologne, Cologne, Germany.
11. Immunogenomics & Neurodegeneration, German Center for Neurodegenerative Diseases (DZNE), Bonn, Germany

*contributed equally

[#]Correspondence:

Jan Rybniker (MD-PhD); Division of Infectious Diseases; University Hospital Cologne
Kerpener Str. 62, 50937 Cologne / Germany;
Email: jan.rybniker@uk-koeln.de
Tel.: +49221 478 89611

Thomas Ulas (PhD); Genomics and Immunoregulation; Life & Medical Sciences (LIMES) Institute,
Carl-Troll-Straße 31, 53115 Bonn / Germany
Email: t.ulas@uni-bonn.de
Tel.: +49 228 433 02641

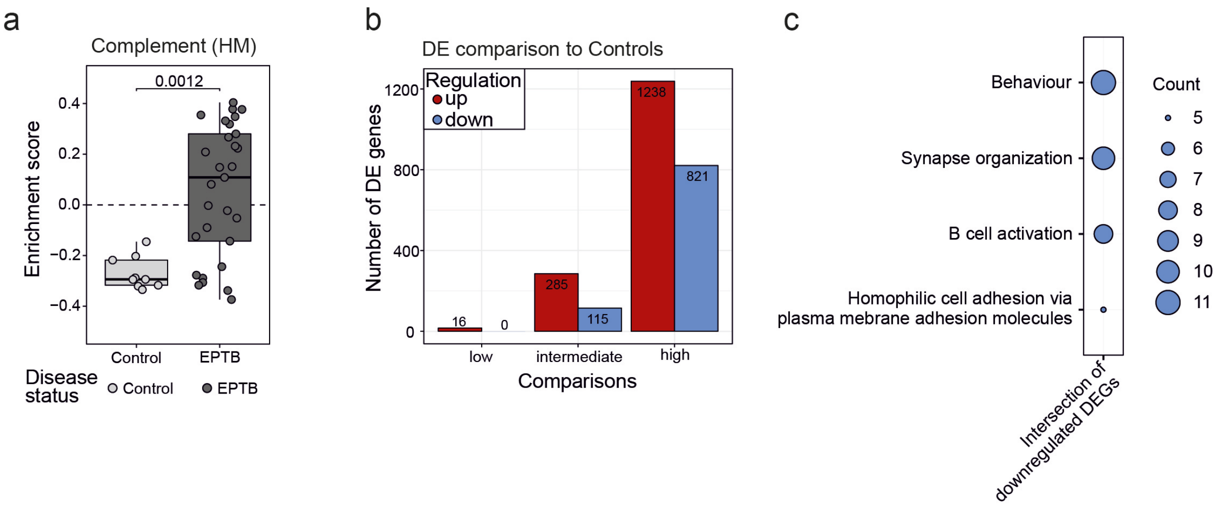

Supplementary Fig. 1: a) Boxplot of gene set variation analysis (GSVA) enrichment scores (ES) of the Molecular Signature Database (MSigDB) Hallmark gene set 'Complement' colored by diseases status. Boxplot show the 25%, 50% (median) and 75% percentile, whiskers denote 1.5 times the interquartile range. Statistics were computed by an unpaired two-sided Wilcoxon test. Control n=9 donors, EPTB n=29 donors. Source data are provided as a Source Data file. **b)** Bar plot of differentially expressed genes (DEGs) (absolute (FC) ≥ 2, Benjamini-Hochberg-adjusted p-value < 0.05) identified between the respective EPTB immunotypes and healthy controls colored by the mode of regulation. **c)** Dot plot of functional enrichment of the intersection of downregulated genes between INF_{int} and INF_{high} using the Gene Ontology (GO) biological processes data base. Dots are scaled based the gene count and colored based on the mode of regulation. Terms with a Benjamini-Hochberg-adjusted p-value < 0.05 were defined as significant.

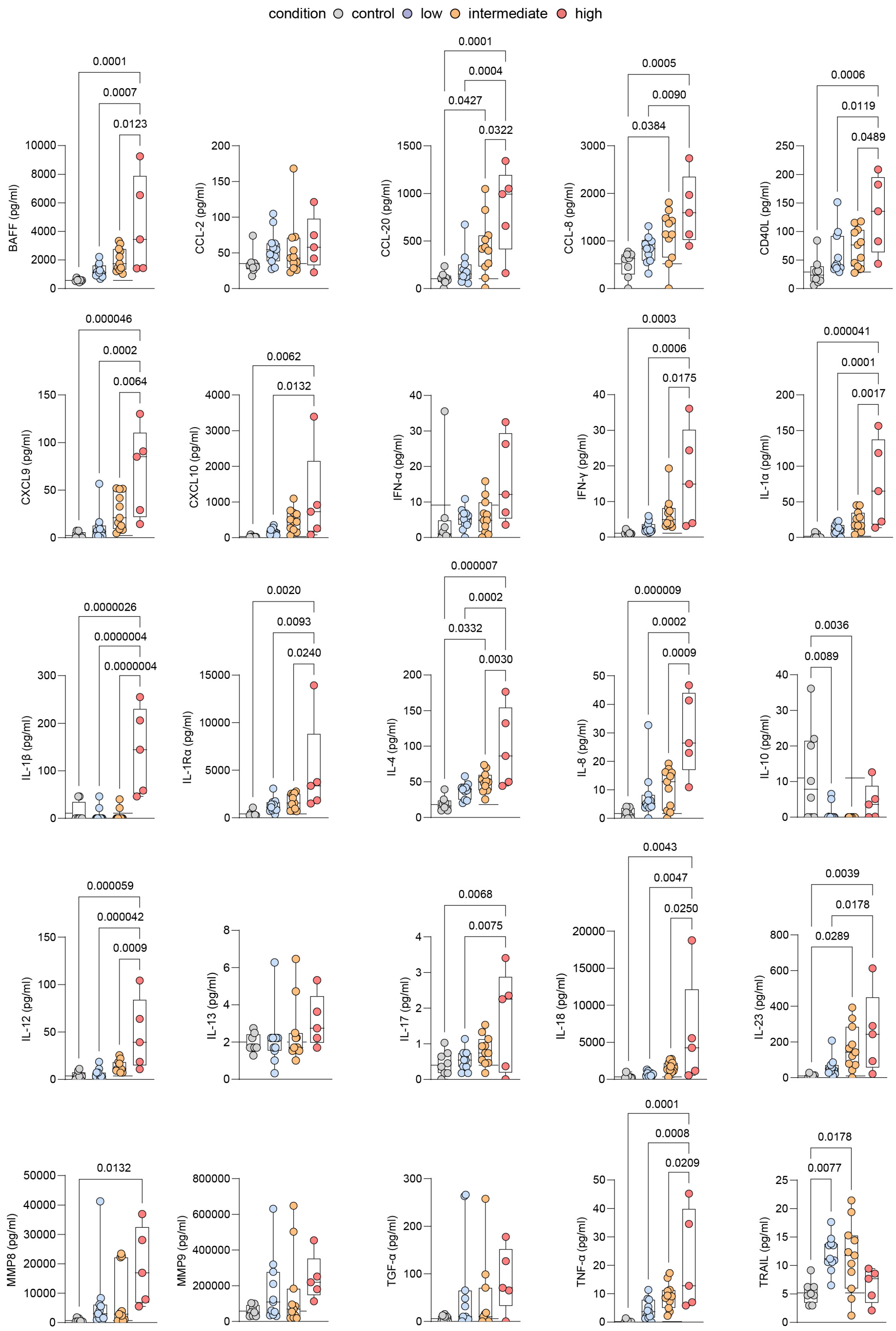
condition control low intermediate high
BAFF (pg/ml)
0.0001
0.0007
0.0123
CCL-2 (pg/ml)
CCL-20 (pg/ml)
0.0001
0.0004
0.0427
0.0322
CCL-8 (pg/ml)
0.0005
0.0090
0.0384
CD40L (pg/ml)
0.0006
0.0119
0.0489
CXCL9 (pg/ml)
0.000046
0.0002
0.0064
CXCL10 (pg/ml)
0.0062
0.0132
IFN-α (pg/ml)
IFN-γ (pg/ml)
0.0003
0.0006
0.0175
IL-1α (pg/ml)
0.000041
0.0001
0.0017
IL-1β (pg/ml)
0.0000026
0.0000004
0.0000004
IL-1Rα (pg/ml)
0.0020
0.0093
0.0240
IL-4 (pg/ml)
0.000007
0.0002
0.0332
0.0030
IL-8 (pg/ml)
0.000009
0.0002
0.0009
IL-10 (pg/ml)
0.0036
0.0089
IL-12 (pg/ml)
0.000059
0.000042
0.0009
IL-13 (pg/ml)
IL-17 (pg/ml)
0.0068
0.0075
IL-18 (pg/ml)
0.0043
0.0047
0.0250
IL-23 (pg/ml)
0.0039
0.0178
0.0289
MMP8 (pg/ml)
0.0132
MMP9 (pg/ml)
TGF-α (pg/ml)
TNF-α (pg/ml)
0.0001
0.0008
0.0209
TRAIL (pg/ml)
0.0178
0.0077

Supplementary Fig. 2: Box plots of plasma cytokine and chemokine bead-array based measurements of all EPTB patients and controls. Concentration depicted in pg/ml. 1-way-ANOVA with Turkey´s post-test derived p-values are indicated. Boxplots show the 25%, 50% (median) and 75% percentile, whiskers range up to the lowest/highest points. Source data are provided as a Source Data file.

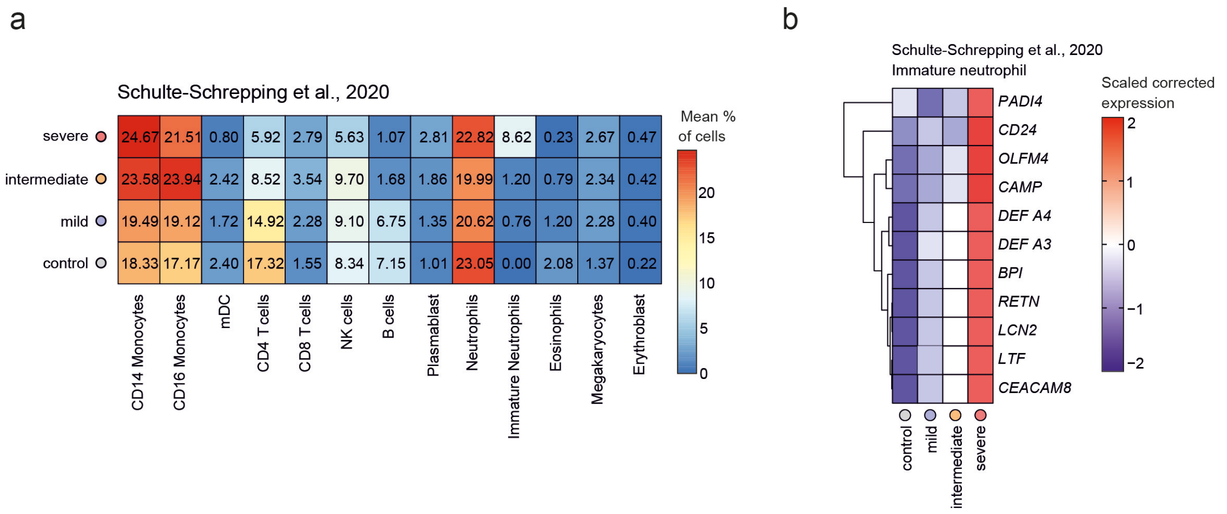

Supplementary Fig. 3: a) Heatmap of the mean percentage of cells computed by cell type deconvolution based on a whole blood reference of healthy individuals from Schulte-Schrepping et al. [1] split by EPTB immunotypes. Only cell types with a fraction above 0 are displayed. **b)** Heatmap of mean scaled, batch-corrected immature neutrophil marker gene expression from Schulte-Schrepping et al. [1] split by EPTB immunotypes.

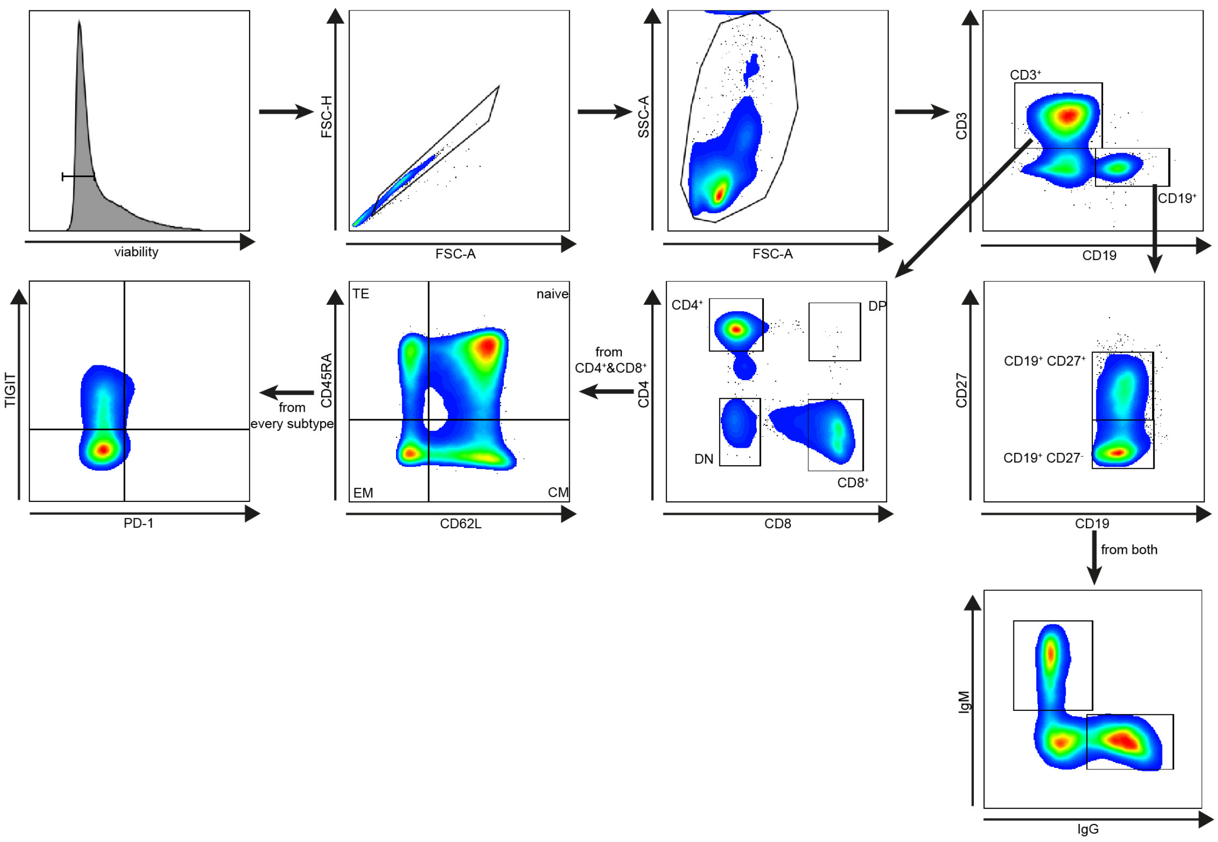

Supplementary Fig. 4: Representative gating example of panel 1.

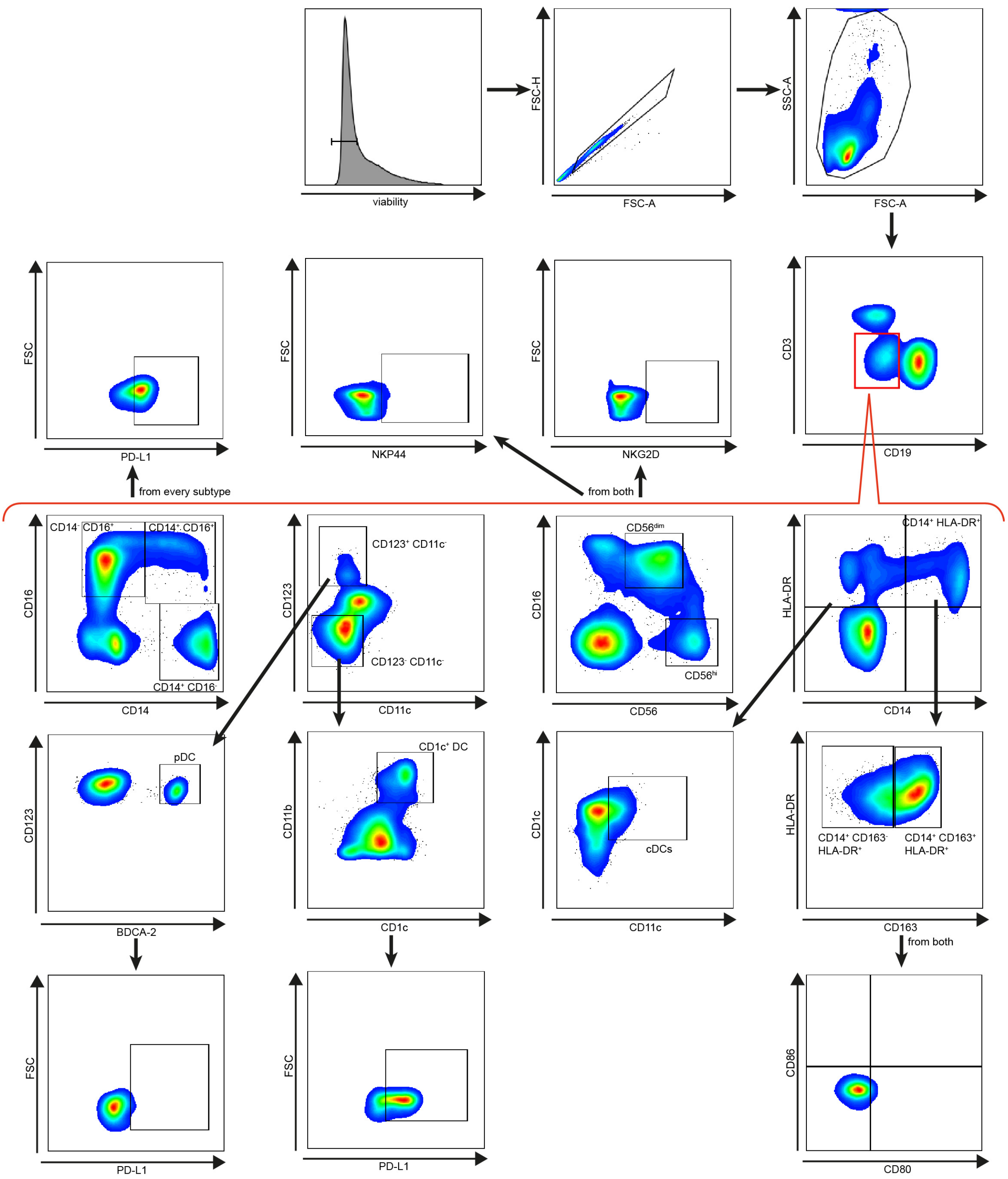

Supplementary Fig. 5: Representative gating example of panel 2.

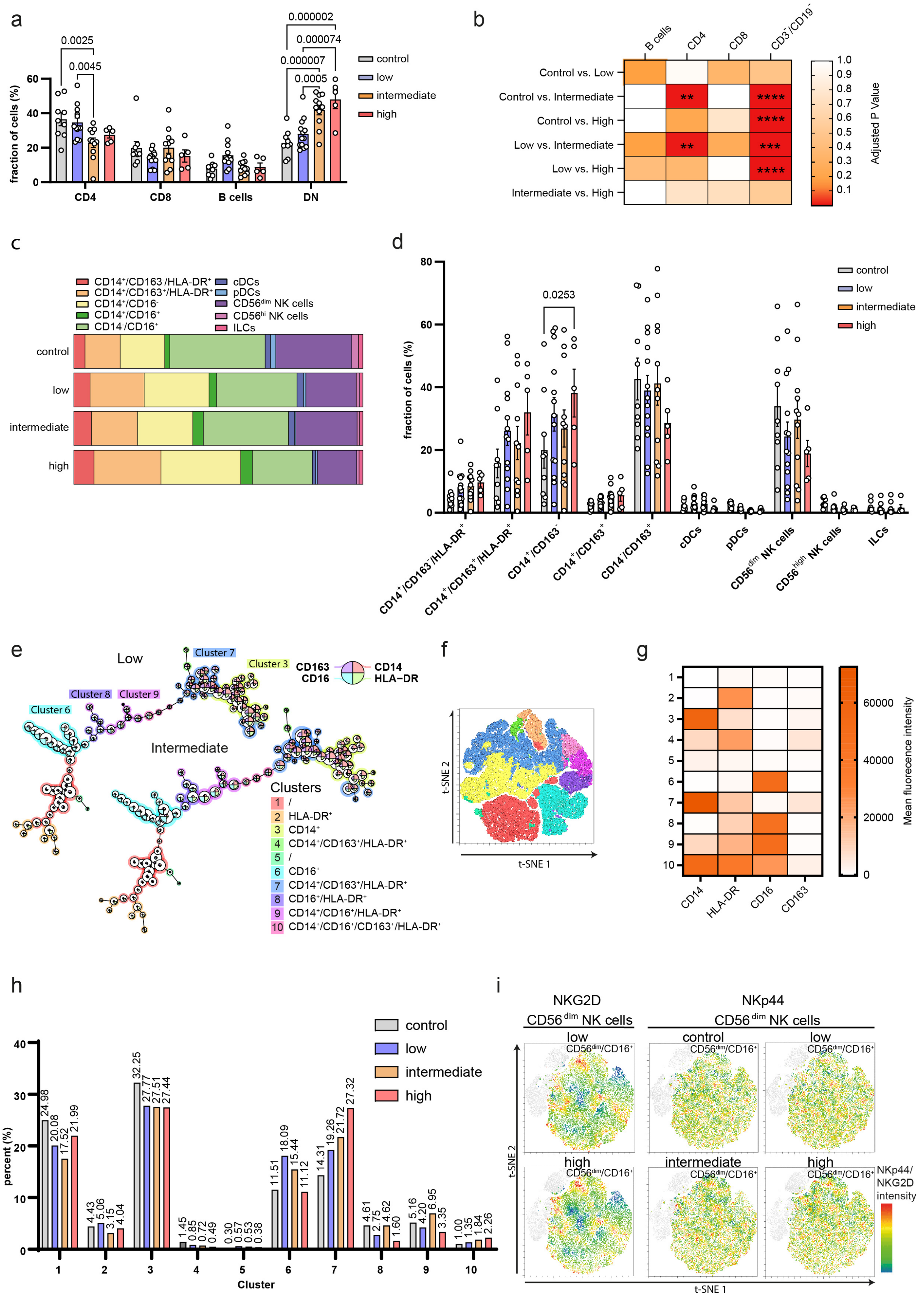
a
0.0025
0.0045
0.000002
0.000074
0.000007
0.0005
fraction of cells (%)
0
20
40
60
CD4
CD8
B cells
DN
control
low
intermediate
high
b
B cells
CD4
CD8
CD3-/CD19-
Control vs. Low
Control vs. Intermediate
Control vs. High
Low vs. Intermediate
Low vs. High
Intermediate vs. High
**
****
****
**
***
****
1.0
0.9
0.8
0.7
0.6
0.5
0.4
0.3
0.2
0.1
Adjusted P Value
c
CD14+/CD163-/HLA-DR+
CD14+/CD163+/HLA-DR+
CD14+/CD16-
CD14+/CD16+
CD14-/CD16+
cDCs
pDCs
CD56dim NK cells
CD56hi NK cells
ILCs
control
low
intermediate
high
d
0.0253
fraction of cells (%)
0
20
40
60
80
control
low
intermediate
high
CD14+/CD163-/HLA-DR+
CD14+/CD163+/HLA-DR+
CD14+/CD163-
CD14+/CD163+
CD14-/CD163+
cDCs
pDCs
CD56dim NK cells
CD56high NK cells
ILCs
e
Low
Intermediate
Cluster 7
Cluster 3
Cluster 8
Cluster 9
Cluster 6
CD163
CD16
CD14
HLA−DR
Clusters
1 /
2 HLA-DR+
3 CD14+
4 CD14+/CD163+/HLA-DR+
5 /
6 CD16+
7 CD14+/CD163+/HLA-DR+
8 CD16+/HLA-DR+
9 CD14+/CD16+/HLA-DR+
10 CD14+/CD16+/CD163+/HLA-DR+
f
t-SNE 2
t-SNE 1
g
1
2
3
4
5
6
7
8
9
10
CD14
HLA-DR
CD16
CD163
60000
40000
20000
0
Mean fluorescence intensity
h
control
low
intermediate
high
percent (%)
0
10
20
30
40
24.98
20.08
17.52
21.99
4.43
5.06
3.15
4.04
32.25
27.77
27.51
27.44
1.45
0.85
0.72
0.49
0.30
0.57
0.53
0.38
11.51
18.09
15.44
11.12
14.31
19.26
21.72
27.32
4.61
2.75
4.62
1.60
5.16
4.20
6.95
3.35
1.00
1.35
1.84
2.26
1
2
3
4
5
6
7
8
9
10
Cluster
i
NKG2D
CD56 dim NK cells
NKp44
CD56 dim NK cells
low
control
low
high
intermediate
high
CD56dim/CD16+
t-SNE 2
t-SNE 1
NKp44/
NKG2D
intensity

Supplementary Fig. 6: a) Individual patient data for the changes in PBMC-subtype ratios in EPTB patients compared to healthy controls, displayed in Figure 3a. Significances measured with a two-way-ANOVA with Turkey`s post-test. Error bars show SEM. Source data are provided as a Source Data file. **b)** Heatmap of the corresponding p-Values to changes in PBMC-subtype ratios in EPTB patients compared to healthy controls, displayed in a) and Figure 3a. Significances are calculated using a two-way-ANOVA with Turkey´s post-test. **c)** Changes in non-T- or B-cell PBMC-subtype ratios in EPTB patients compared to healthy controls. **d)** Individual patient data for the changes in non-T- or B-cell PBMC-subtype ratios in EPTB patients compared to healthy controls, displayed in c). Significances measured with a 2-way-ANOVA with Turkey`s post-test. Error bars show SEM. Source data are provided as a Source Data file. **e)** FlowSOM minimum spanning trees displaying changes of monocyte and macrophage subtypes in INF_{low} and INF_{int} EPTB patients. Clustering of $CD3^-/CD19^-/CD56^-/CD123^-$ cells based on CD14, CD16, CD163 and HLA-DR expression. **f)** 2-D projection of the flowSOM clusters displayed in e) onto a t-SNE plot of monocyte and macrophage subtypes in all patients and controls. T-SNE calculated based on the same markers as the FlowSOM displayed in e). **g)** Heatmap of the relative marker-expression within the monocyte/macrophage FlowSOM clusters displayed in e). Mean fluorescence intensity is displayed. **h)** Fractions of the different monocyte/macrophage FlowSOM clusters displayed in e) within the different patient groups and healthy controls. **i)** T-SNE of $CD56^+$ cells displaying relative NKG2D and NKp44 expression in CD56dim NK cells of INF_{low} and INF_{high} EPTB patients, or all control and patient groups, respectively. T-SNE calculated based on NKP44, NKG2D, CD94, CD16 and CD56 expression.

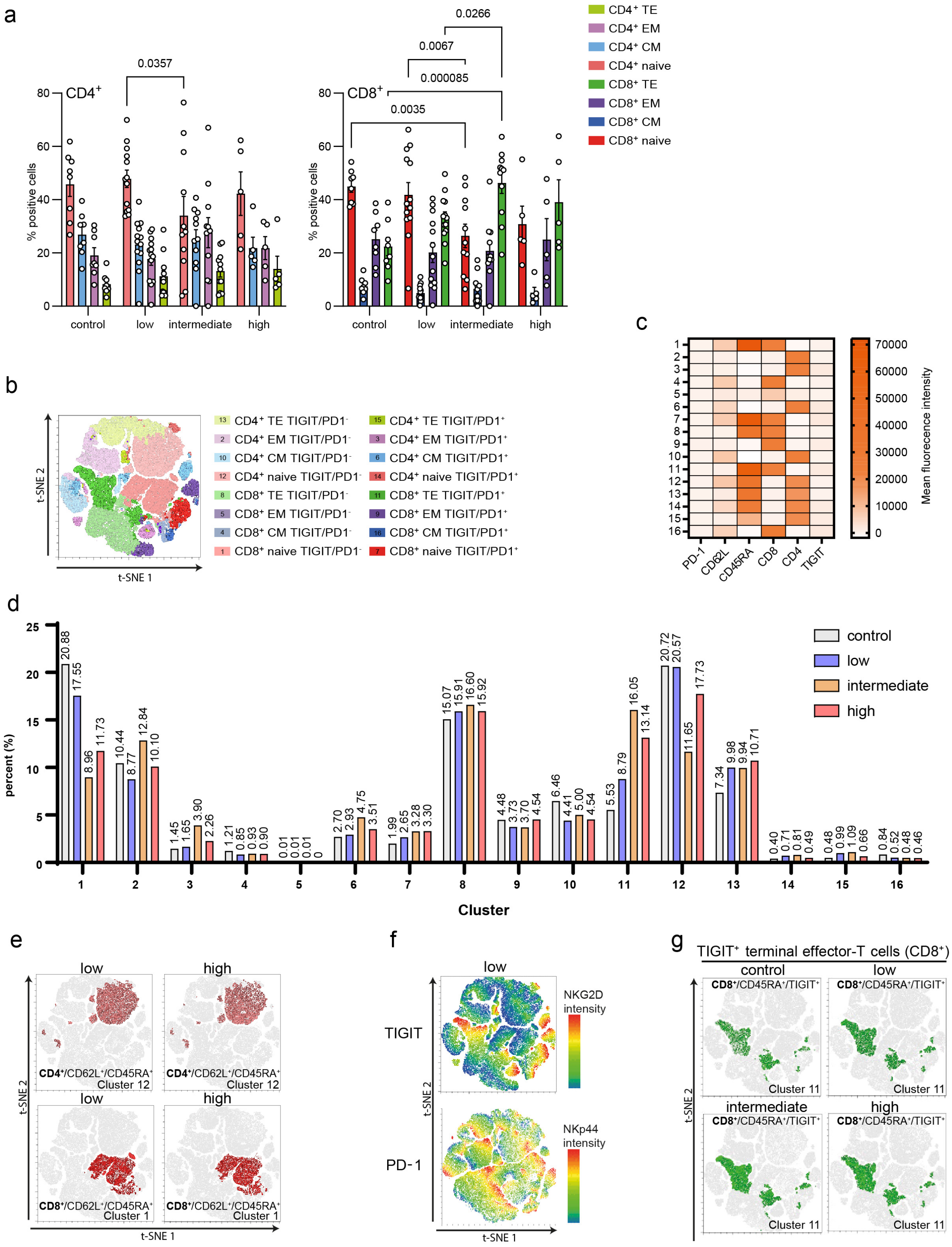

a
CD4+ TE
CD4+ EM
CD4+ CM
CD4+ naive
CD8+ TE
CD8+ EM
CD8+ CM
CD8+ naive
CD4+
CD8+
% positive cells
0.0357
0.0266
0.0067
0.000085
0.0035
control
low
intermediate
high
b
t-SNE 1
t-SNE 2
13 CD4+ TE TIGIT/PD1-
15 CD4+ TE TIGIT/PD1+
2 CD4+ EM TIGIT/PD1-
3 CD4+ EM TIGIT/PD1+
10 CD4+ CM TIGIT/PD1-
6 CD4+ CM TIGIT/PD1+
12 CD4+ naive TIGIT/PD1-
14 CD4+ naive TIGIT/PD1+
8 CD8+ TE TIGIT/PD1-
11 CD8+ TE TIGIT/PD1+
5 CD8+ EM TIGIT/PD1-
9 CD8+ EM TIGIT/PD1+
4 CD8+ CM TIGIT/PD1-
16 CD8+ CM TIGIT/PD1+
1 CD8+ naive TIGIT/PD1-
7 CD8+ naive TIGIT/PD1+
c
PD-1
CD62L
CD45RA
CD8
CD4
TIGIT
Mean fluorescence intensity
70000
60000
50000
40000
30000
20000
10000
0
d
percent (%)
Cluster
control
low
intermediate
high
e
low
high
CD4+/CD62L+/CD45RA+ Cluster 12
CD8+/CD62L+/CD45RA+ Cluster 1
t-SNE 1
t-SNE 2
f
low
TIGIT
PD-1
NKG2D intensity
NKp44 intensity
t-SNE 1
t-SNE 2
g
TIGIT+ terminal effector-T cells (CD8+)
control
low
intermediate
high
CD8+/CD45RA+/TIGIT+
Cluster 11
t-SNE 2

Supplementary Fig. 7: a) Individual patient data of the T- cell subtype distribution in $CD4^+$ and $CD8^+$ T cells of healthy controls and EPTB patients. Significances measured with a 2-way-ANOVA with Turkey`s post-test. Error bars show SEM. Source data are provided as a Source Data file. **b)** 2-D projection of the flowSOM clusters displayed in Figure 3h and 3i onto a t-SNE plot of T cells in all patients and controls. T-SNE calculated based on the markers listed in c) **c)** Heatmap of the relative marker-expression within the T cell FlowSOM clusters displayed in b) and Figure 3. Mean fluorescence intensity is displayed. **d)** Fractions of the T cell FlowSOM clusters displayed in b), c) and Figure 3 within the different patient groups and healthy controls. **e)** T-SNE plots of $CD4^+$ and $CD8^+$ cells highlighting the proportion of naive T cells in INF_{low} and INF_{high} EPTB patients. T-SNE calculated based on CD4, CD8, CD62L, CD45RA, PD-1 and TIGIT expression. **f)** T-SNE of $CD4^+$ and $CD8^+$ cells displaying relative TIGIT and PD-1 expression in T cells of INF_{low} EPTB patients. PD-1 t-SNE (bottom) calculated based on CD4, CD8, CD62L, CD45RA and PD-1 expression. **g)** T-SNE plots of t cells highlighting the proportion of $TIGIT^+$ $CD8^+$ terminal effector t cells in healthy controls and EPTB patients. T-SNE calculated based on CD4, CD8, CD62L, CD45RA, PD-1 and TIGIT expression.

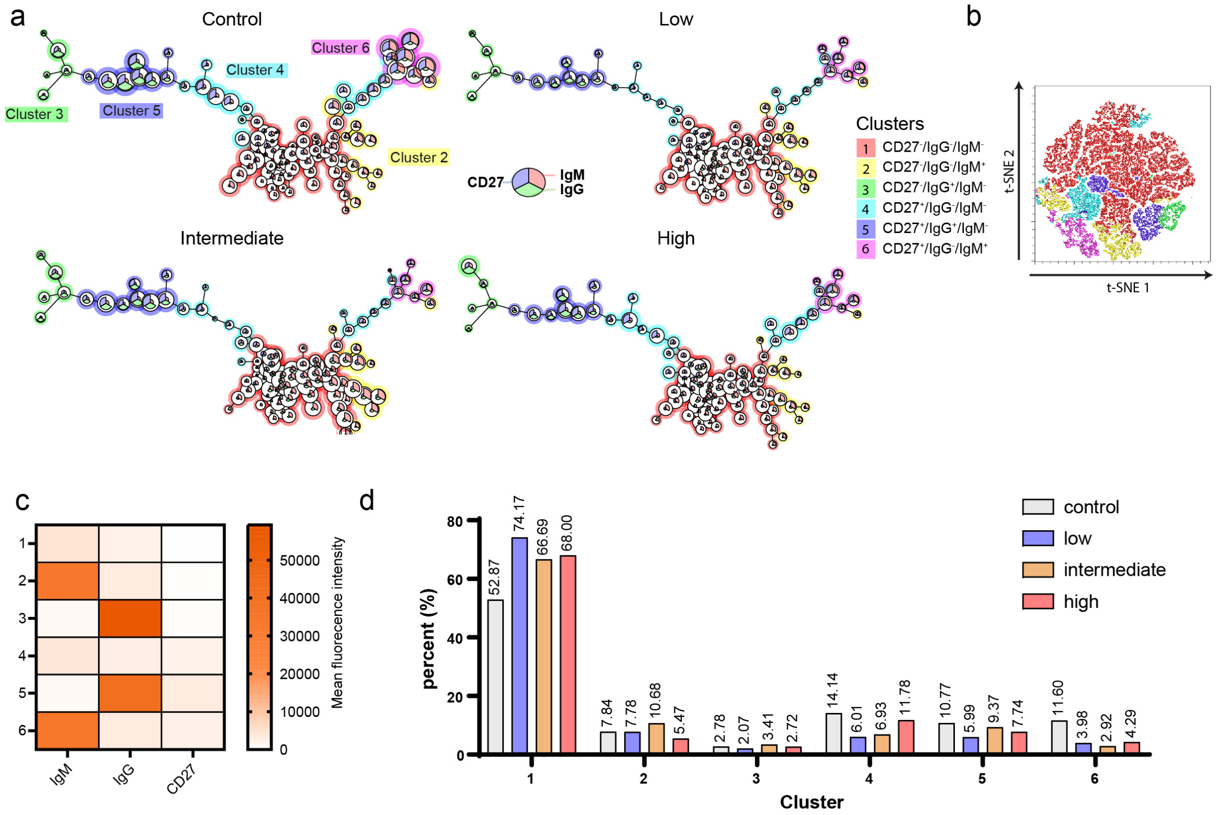

Supplementary Fig. 8: a) FlowSOM minimum spanning trees displaying changes of B cell subtypes in EPTB patients and healthy controls. Clustering of CD19$^+$ cells based on CD27, IgM and IgG expression. **b)** 2-D projection of the flowSOM clusters displayed in a) onto a t-SNE plot of B-cell subtypes in all patients and controls. T-SNE calculated based on the same markers as the FlowSOM displayed in a). **c)** Heatmap of the relative marker-expression within the B cell FlowSOM clusters displayed in a). Mean fluorescence intensity is displayed. **d)** Fractions of the different B cell FlowSOM clusters displayed in a) within the different patient groups and healthy controls.

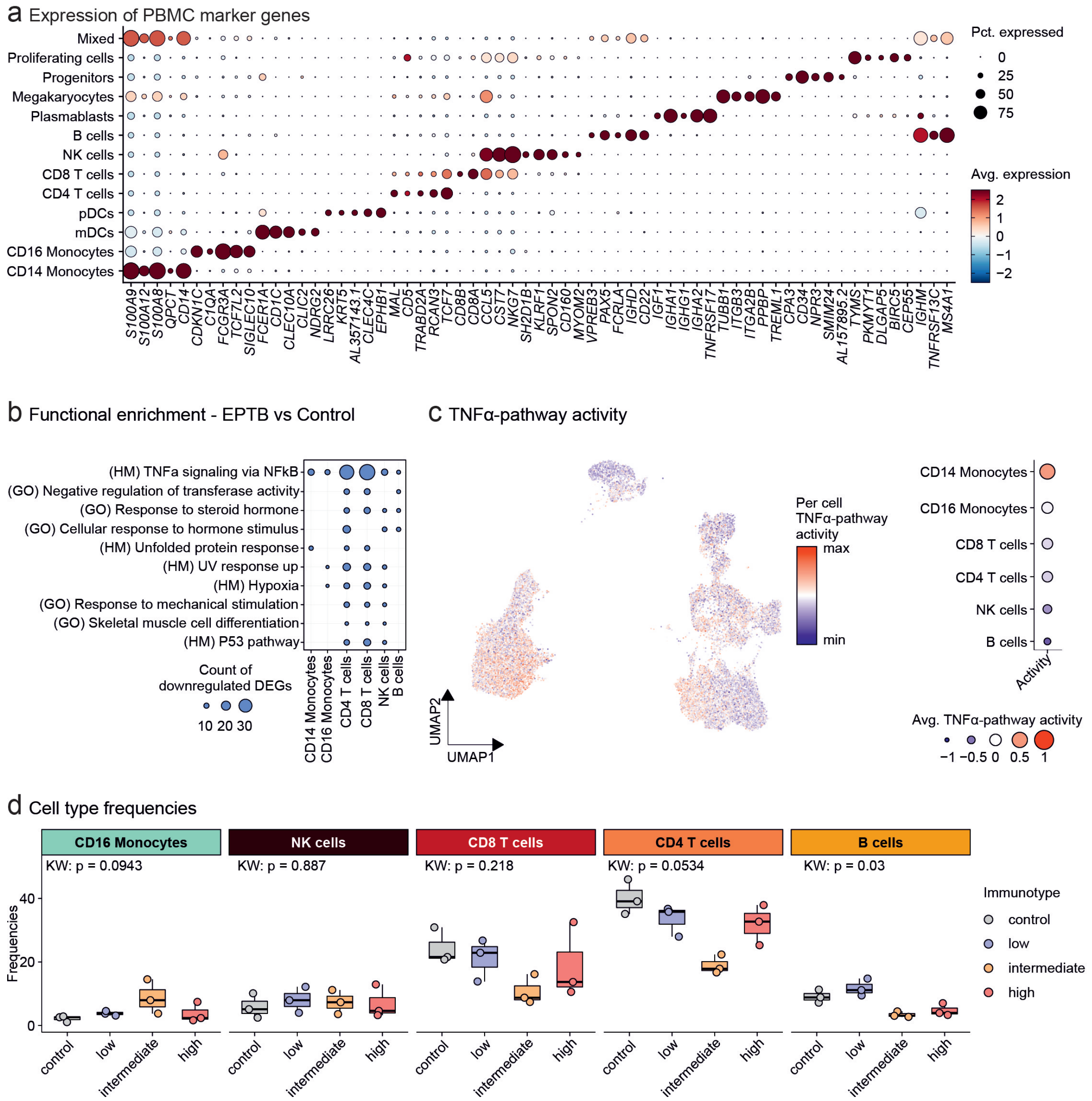

Supplementary Fig. 9: a) Dot plot of marker gene expression for all detected cell types within scRNA-seq data. Dot size indicates percentage of expression per cell state, color indicates scaled average expression. **b)** Dot plot of functional enrichment of upregulated genes in cell types with more than 2,000 cells identified in EPTB patients using the GO biological processes, KEGG and MSigDB Hallmark gene set databases. Only top terms ordered by adjusted p-value that were significantly enriched in at least three comparisons are displayed (Benjamini-Hochberg-adjustedp-value <0.05) **c)** UMAP visualization of the PBMCs colored by PROGENy-inferred TNF-α pathway activity score. Median activity scores per cell types with more than 2,000 cells are indicated as dots scaled and colored by score. **d)** Boxplot of percentage of cells for controls and EPTB patients per selected cell types with more than 2,000 cells. Boxplots show the 25%, 50% (median) and 75% percentile, whiskers denote 1.5 times the interquartile

range. Statistics were computed by Kruskal-Wallis test. For each group: n=3 donors. Source data are provided as a Source Data file.

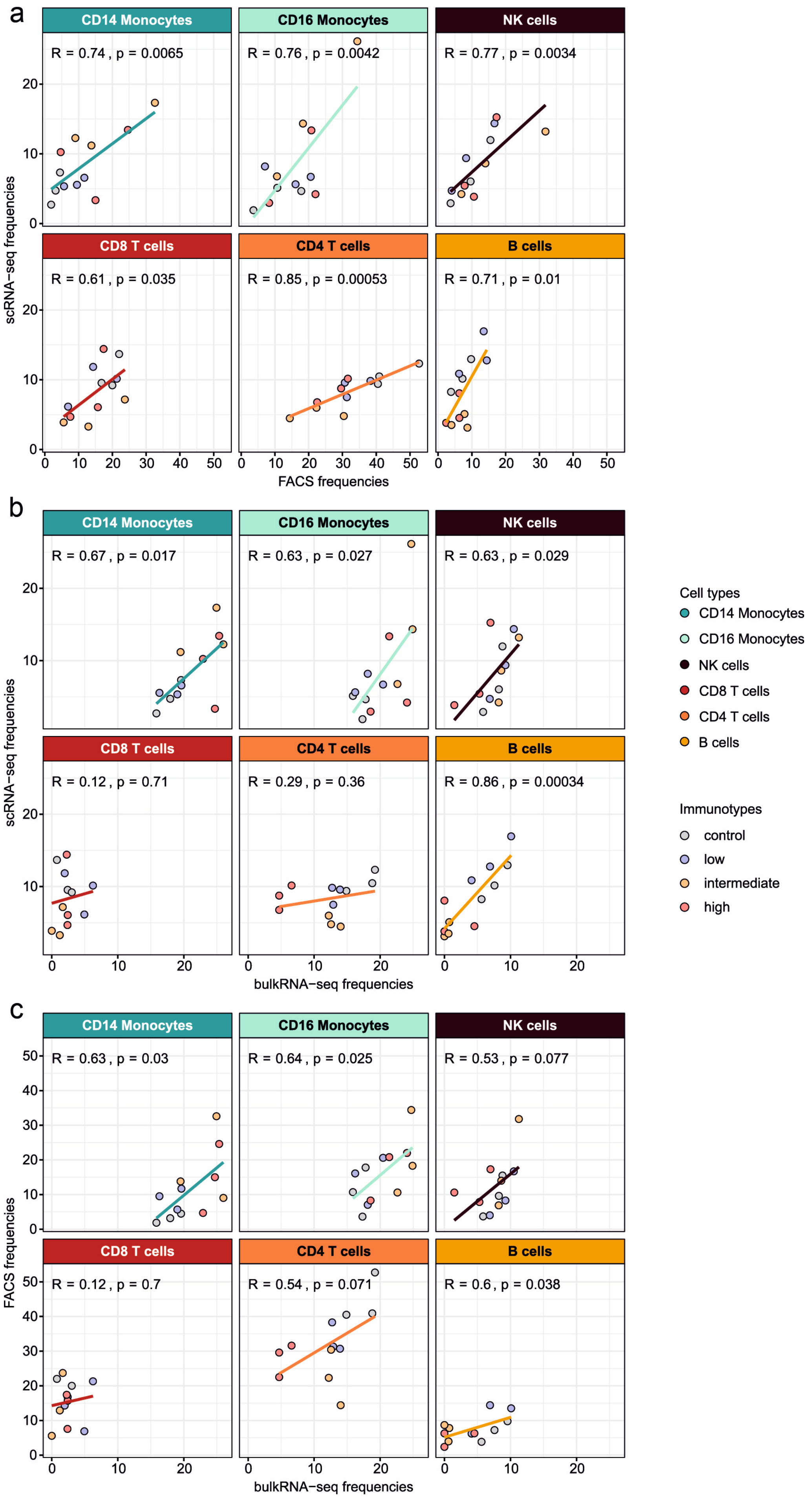

a
CD14 Monocytes
R = 0.74 , p = 0.0065
CD16 Monocytes
R = 0.76 , p = 0.0042
NK cells
R = 0.77 , p = 0.0034
CD8 T cells
R = 0.61 , p = 0.035
CD4 T cells
R = 0.85 , p = 0.00053
B cells
R = 0.71 , p = 0.01
scRNA−seq frequencies
FACS frequencies
b
CD14 Monocytes
R = 0.67 , p = 0.017
CD16 Monocytes
R = 0.63 , p = 0.027
NK cells
R = 0.63 , p = 0.029
CD8 T cells
R = 0.12 , p = 0.71
CD4 T cells
R = 0.29 , p = 0.36
B cells
R = 0.86 , p = 0.00034
scRNA−seq frequencies
bulkRNA−seq frequencies
c
CD14 Monocytes
R = 0.63 , p = 0.03
CD16 Monocytes
R = 0.64 , p = 0.025
NK cells
R = 0.53 , p = 0.077
CD8 T cells
R = 0.12 , p = 0.7
CD4 T cells
R = 0.54 , p = 0.071
B cells
R = 0.6 , p = 0.038
FACS frequencies
bulkRNA−seq frequencies
Cell types
CD14 Monocytes
CD16 Monocytes
NK cells
CD8 T cells
CD4 T cells
B cells
Immunotypes
control
low
intermediate
high

Supplementary Fig. 10: a) Scatter plot of frequencies of major cell types (present in all modalities) as measured by FACS and scRNA-seq data of matching patients colored based on the EPTB immunotypes. Effect sizes were computed using the Pearson's correlation coefficient *r.* For each group: n=3 donors. Source data are provided as a Source Data file. **b)** Scatter plot of frequencies of major cell types (present in all modalities) as estimated in bulk RNA-seq data and measured by scRNA-seq data of matching patients colored based on the EPTB immunotypes. Effect sizes were computed using the Pearson's correlation coefficient *r.* For each group: n=3 donors. Source data are provided as a Source Data file. **c)** Scatter plot of frequencies of major cell types (present in all modalities) as estimated in bulk RNA-seq data and measured by FACS-seq data of matching patients colored based on the EPTB immunotypes. Effect sizes were computed using the Pearson's correlation coefficient *r.* For each group: n=3 donors. Source data are provided as a Source Data file.

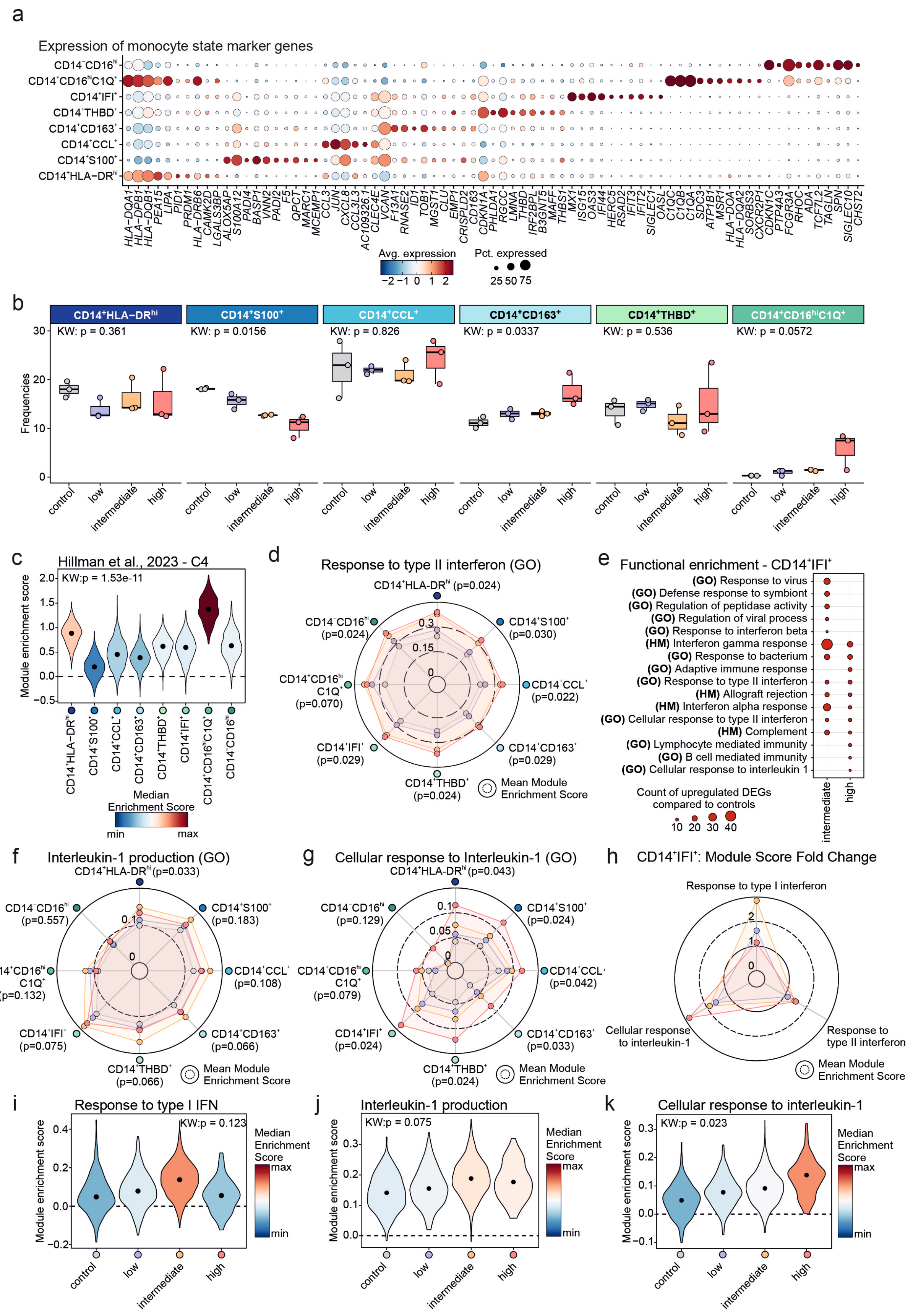
a
Expression of monocyte state marker genes
CD14⁻CD16hi
CD14+CD16hiC1Q+
CD14+IFI+
CD14+THBD+
CD14+CD163+
CD14+CCL+
CD14+S100+
CD14+HLA-DRhi
HLA-DQA1 HLA-DPB1 HLA-DQB1 PEA15 LIPA PID1 PRDM1 HLA-DRB6 CAMK2D LGALS3BP ALOX5AP S100A12 PADI4 BASP1 VNN2 PADI2 F5 QPCT MARC1 MCEMP1 CCL3 JUN CXCL8 CCL3L3 AC109326.1 CLEC4E VCAN F13A1 RNASE2 ID1 TOB1 MGST1 CLU EMP1 CRISPLD2 CD163 CDKN1A PHLDA1 RGCC LMNA THBD IRF2BPL B3GNT5 MAFF THBS1 MX1 ISG15 OAS3 IFI44 HERC5 RSAD2 IFIT3 IFIT2 SIGLEC1 OASL C1QC C1QB C1QA SDC3 ATP1B1 MSR1 HLA-DOA HLA-DQA2 SORBS3 CXCR2P1 CDKN1C PTP4A3 FCGR3A RHOC ADA TCF7L2 TAGLN SPN SIGLEC10 CHST2
Avg. expression
−2 −1 0 1 2
Pct. expressed
25 50 75
b
CD14+HLA-DRhi
KW: p = 0.361
CD14+S100+
KW: p = 0.0156
CD14+CCL+
KW: p = 0.826
CD14+CD163+
KW: p = 0.0337
CD14+THBD+
KW: p = 0.536
CD14+CD16hiC1Q+
KW: p = 0.0572
Frequencies
0 10 20 30
control low intermediate high
c
Hillman et al., 2023 - C4
KW:p = 1.53e-11
Module enrichment score
−0.5 0.0 0.5 1.0 1.5 2.0
CD14+HLA-DRhi CD14+S100+ CD14+CCL+ CD14+CD163+ CD14+THBD+ CD14+IFI+ CD14+CD16hiC1Q+ CD14⁻CD16hi
Median Enrichment Score
min max
d
Response to type II interferon (GO)
CD14+HLA-DRhi (p=0.024)
CD14+S100+ (p=0.030)
CD14+CCL+ (p=0.022)
CD14+CD163+ (p=0.029)
CD14+THBD+ (p=0.024)
CD14+IFI+ (p=0.029)
CD14+CD16hi C1Q+ (p=0.070)
CD14⁻CD16hi (p=0.024)
0 0.15 0.3
Mean Module Enrichment Score
e
Functional enrichment - CD14+IFI+
(GO) Response to virus
(GO) Defense response to symbiont
(GO) Regulation of peptidase activity
(GO) Regulation of viral process
(GO) Response to interferon beta
(HM) Interferon gamma response
(GO) Response to bacterium
(GO) Adaptive immune response
(GO) Response to type II interferon
(HM) Allograft rejection
(HM) Interferon alpha response
(GO) Cellular response to type II interferon
(HM) Complement
(GO) Lymphocyte mediated immunity
(GO) B cell mediated immunity
(GO) Cellular response to interleukin 1
Count of upregulated DEGs compared to controls
10 20 30 40
intermediate high
f
Interleukin-1 production (GO)
CD14+HLA-DRhi (p=0.033)
CD14+S100+ (p=0.183)
CD14+CCL+ (p=0.108)
CD14+CD163+ (p=0.066)
CD14+THBD+ (p=0.066)
CD14+IFI+ (p=0.075)
CD14+CD16hi C1Q+ (p=0.132)
CD14⁻CD16hi (p=0.557)
0 0.1
Mean Module Enrichment Score
g
Cellular response to Interleukin-1 (GO)
CD14+HLA-DRhi (p=0.043)
CD14+S100+ (p=0.024)
CD14+CCL+ (p=0.042)
CD14+CD163+ (p=0.033)
CD14+THBD+ (p=0.024)
CD14+IFI+ (p=0.024)
CD14+CD16hi C1Q+ (p=0.079)
CD14⁻CD16hi (p=0.129)
0 0.05 0.1
Mean Module Enrichment Score
h
CD14+IFI+: Module Score Fold Change
Response to type I interferon
Cellular response to interleukin-1
Response to type II interferon
0 1 2
Mean Module Enrichment Score
i
Response to type I IFN
KW:p = 0.123
Module enrichment score
−0.2 0.0 0.2 0.4
Median Enrichment Score
max min
control low intermediate high
j
Interleukin-1 production
KW:p = 0.075
Module enrichment score
0.0 0.1 0.2 0.3
Median Enrichment Score
max min
control low intermediate high
k
Cellular response to interleukin-1
KW:p = 0.023
Module enrichment score
−0.1 0.0 0.1 0.2 0.3
Median Enrichment Score
max min
control low intermediate high

Supplementary Fig. 11: a) Dot plot of marker gene expression for monocyte states within scRNA-seq data. Dot size indicates percentage of expression per cell state, color indicates scaled average expression. **b)** Boxplot of percentage of cells for controls and EPTB patients per selected cell state. Boxplots show the 25%, 50% (median) and 75% percentile, whiskers denote 1.5 times the interquartile range. Statistics were computed by Kruskal-Wallis test. **c)** Violin plot of module ES of the top 20 marker genes of the C4 cluster published in Hillman et al [2]. colored by median enrichment score and split by monocyte states. **d)** Radar plot of module ES of the GO term 'response to type II interferon' per monocyte state. Dots represent the median ES per EPTB immunotype and are colored accordingly. Statistics were computed with a Kruskal-Wallis-test.. **e)** Dot plot of functional enrichment of upregulated genes in $CD14^{+}IFI^{+}$ monocytes between EPTB patients and healthy controls using the GO biological processes, KEGG and MSigDB gene set databases. The top 10 terms significantly enriched per comparison (p-value <0.05) are displayed ordered by Benjamini-Hochberg-adjusted p-value. **f,g)** Radar plot of median module ES of the the combination of the GO terms 'cellular response to interleukin-1' and 'response to interleukin-1'as well as the GO term 'interleukin-1 production' per monocyte state, respectively. Dots represent the median ES per EPTB immunotype and are colored accordingly. Statistics were computed with a Kruskal-Wallis-test. **h)** Radar plot of median module ES fold changes of the GO terms 'response to type I IFN', 'response to type II IFN' as well as a combination of 'cellular response to interleukin-1' and 'response to interleukin-1' between EPTB patients and healthy controls in $CD14^{+}IFI^{+}$ monocytes. Dots are colored based on the EPTB immunotypes and represent the mean enrichment of donors.. **i-k)** Violin plots of the ES from $CD14^{+}IFI^{+}$ cells per EPTB subtype and healthy controls across cell subtypes. Median ES are indicated as black dots. Kruskal-Wallis-test derived p-values per signature enrichment per cell subtype are indicated. For each group: n=3 donors for the respective figures. Source data are provided as a Source Data file for the respective figures.

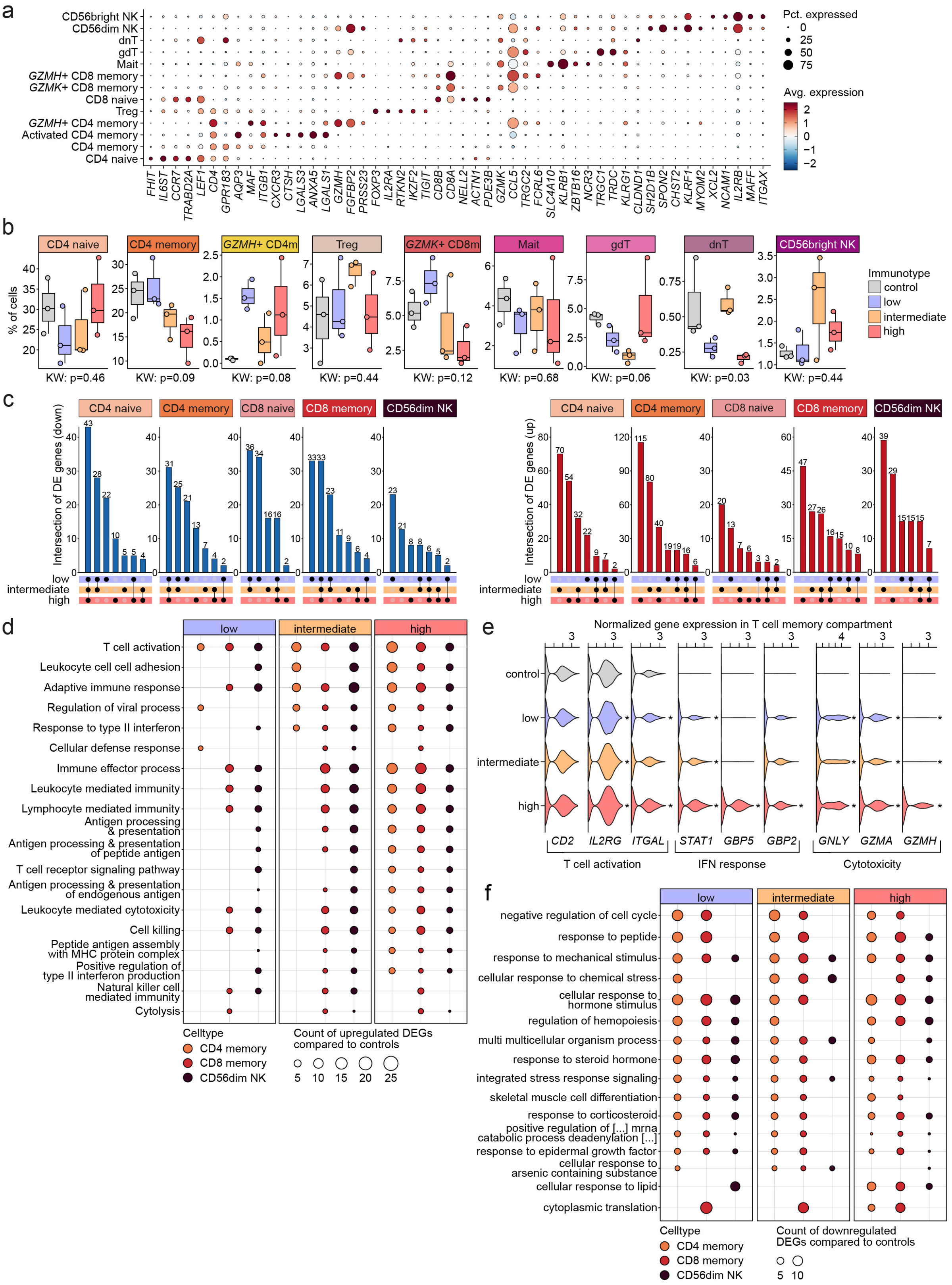

a
CD56bright NK
CD56dim NK
dnT
gdT
Mait
GZMH+ CD8 memory
GZMK+ CD8 memory
CD8 naive
Treg
GZMH+ CD4 memory
Activated CD4 memory
CD4 memory
CD4 naive
FHIT IL6ST CCR7 TRABD2A LEF1 CD4 GPR183 AQP3 MAF ITGB1 CXCR3 CTSH LGALS3 ANXA5 LGALS1 GZMH FGFBP2 PRSS23 FOXP3 IL2RA RTKN2 IKZF2 TIGIT CD8B CD8A NELL2 ACTN1 PDE3B GZMK CCL5 TRGC2 FCRL6 SLC4A10 KLRB1 ZBTB16 NCR3 TRGC1 TRDC KLRG1 CLDND1 SH2D1B SPON2 CHST2 KLRF1 MYOM2 XCL2 NCAM1 IL2RB MAFF ITGAX
Pct. expressed
0
25
50
75
Avg. expression
2
1
0
−1
−2
b
CD4 naive
CD4 memory
GZMH+ CD4m
Treg
GZMK+ CD8m
Mait
gdT
dnT
CD56bright NK
% of cells
KW: p=0.46
KW: p=0.09
KW: p=0.08
KW: p=0.44
KW: p=0.12
KW: p=0.68
KW: p=0.06
KW: p=0.03
KW: p=0.44
Immunotype
control
low
intermediate
high
c
CD4 naive
CD4 memory
CD8 naive
CD8 memory
CD56dim NK
Intersection of DE genes (down)
Intersection of DE genes (up)
low
intermediate
high
d
low
intermediate
high
T cell activation
Leukocyte cell cell adhesion
Adaptive immune response
Regulation of viral process
Response to type II interferon
Cellular defense response
Immune effector process
Leukocyte mediated immunity
Lymphocyte mediated immunity
Antigen processing & presentation
Antigen processing & presentation of peptide antigen
T cell receptor signaling pathway
Antigen processing & presentation of endogenous antigen
Leukocyte mediated cytotoxicity
Cell killing
Peptide antigen assembly with MHC protein complex
Positive regulation of type II interferon production
Natural killer cell mediated immunity
Cytolysis
Celltype
CD4 memory
CD8 memory
CD56dim NK
Count of upregulated DEGs compared to controls
5 10 15 20 25
e
Normalized gene expression in T cell memory compartment
control
low
intermediate
high
CD2 IL2RG ITGAL STAT1 GBP5 GBP2 GNLY GZMA GZMH
T cell activation
IFN response
Cytotoxicity
f
low
intermediate
high
negative regulation of cell cycle
response to peptide
response to mechanical stimulus
cellular response to chemical stress
cellular response to hormone stimulus
regulation of hemopoiesis
multi multicellular organism process
response to steroid hormone
integrated stress response signaling
skeletal muscle cell differentiation
response to corticosteroid
positive regulation of [...] mrna catabolic process deadenylation [...]
response to epidermal growth factor
cellular response to arsenic containing substance
cellular response to lipid
cytoplasmic translation
Celltype
CD4 memory
CD8 memory
CD56dim NK
Count of downregulated DEGs compared to controls
5 10

Supplementary Fig. 12: scRNA-seq reveals a T and NK cell phenotypic switch in EPTB. **a)** Marker gene expression for cell states in the T and NK cell space from scRNA-seq data. Dot size indicates percentage of expression per cell state, color indicates scaled average expression. **b)** Boxplot of percentage of cells for controls and EPTB patients per selected cell state. Boxplots show the 25%, 50% (median) and 75% percentile, whiskers denote 1.5 times the interquartile range. Statistics were computed by Kruskal-Wallis test. For each group: n=3 donors. Source data are provided as a Source Data file. **c)** Upset plot of the intersection of down- and upregulated differentially expressed (DE) genes from Figure 5c between EPTB subtypes per cell subtype. **d)** Functional enrichment of upregulated genes in memory T cells and CD56^{dim} NK cells identified in EPTB patients using the GO biological processes database. Only top terms ordered by adjusted p-value that were significantly enriched in at least three comparisons are displayed (Benjamini-Hochberg adjusted p-value <0.05). **e)** Violin plots of selected upregulated genes from functional terms from Figure 5e-f in EPTB subtypes and controls in CD4 and CD8 memory T cells. Significantly higher expression in the memory T cell compartment in EPTB is indicated with asterisks (parameters: log2FC = 0.25, min.pct = 0.1, Wilcoxon test, Bonferroni adjusted p-value < 0.05). **f)** Functional enrichment of downregulated genes in memory T cells and CD56^{dim} NK cells identified in EPTB patients using the GO biological processes database. Only top terms ordered by adjusted p-value that were significantly enriched in at least three comparisons are displayed (Benjamini-Hochberg adjusted p-value <0.05).

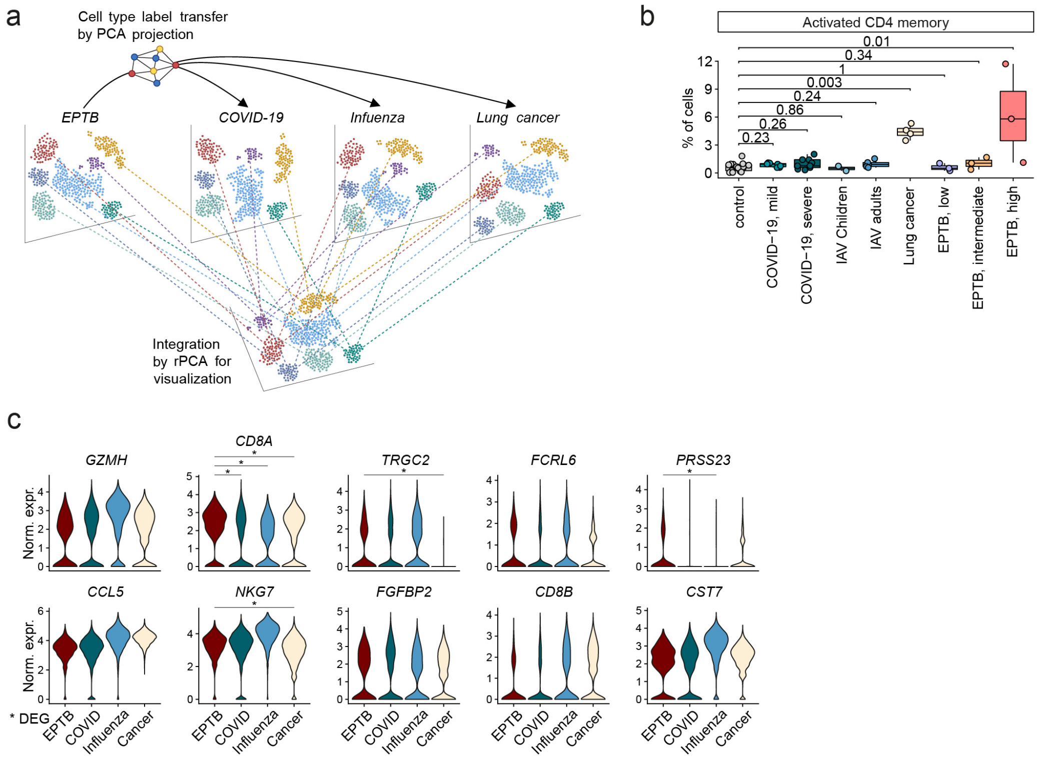

Supplementary Fig. 13: scRNA-seq reveals a T and NK cell phenotypic switch in EPTB. **a)** Scheme of cell state annotation and integration of disease cohorts from Schulte-Schrepping et al.[1], Yanagihara et al. [3], and Zhang et al. [4]. Annotation was performed by Seurat reference mapping label transfer per cohort from the EPTB dataset and integration of all datasets was performed by reciprocal PCA integration for visualization in Figure 5h. Created in BioRender. Spintge, J. (2025) https://BioRender.com/ix2xmc9 **b)** Boxplot of percentage of activated CD4 memory T cells for EPTB and other disease patients. Statistics were computed by an unpaired two-sided Wilcoxon test with Benjamini-Hochberg adjustment. Source data are provided as a Source Data file. **c)** Violin plots of the top marker genes of *GZMH*$^+$ CD8$^+$ memory T cells in EPTB and other disease cohorts. *GZMH*$^+$ CD8$^+$ memory T cells are defined by Seurat reference mapping label transfer from the EPTB dataset. Significantly higher expression in EPTB is indicated with asterisks (parameters: log2FC = 0.25, min.pct = 0.1, unpaired two-sided Wilcoxon test, Bonferroni adjusted p-value < 0.05).

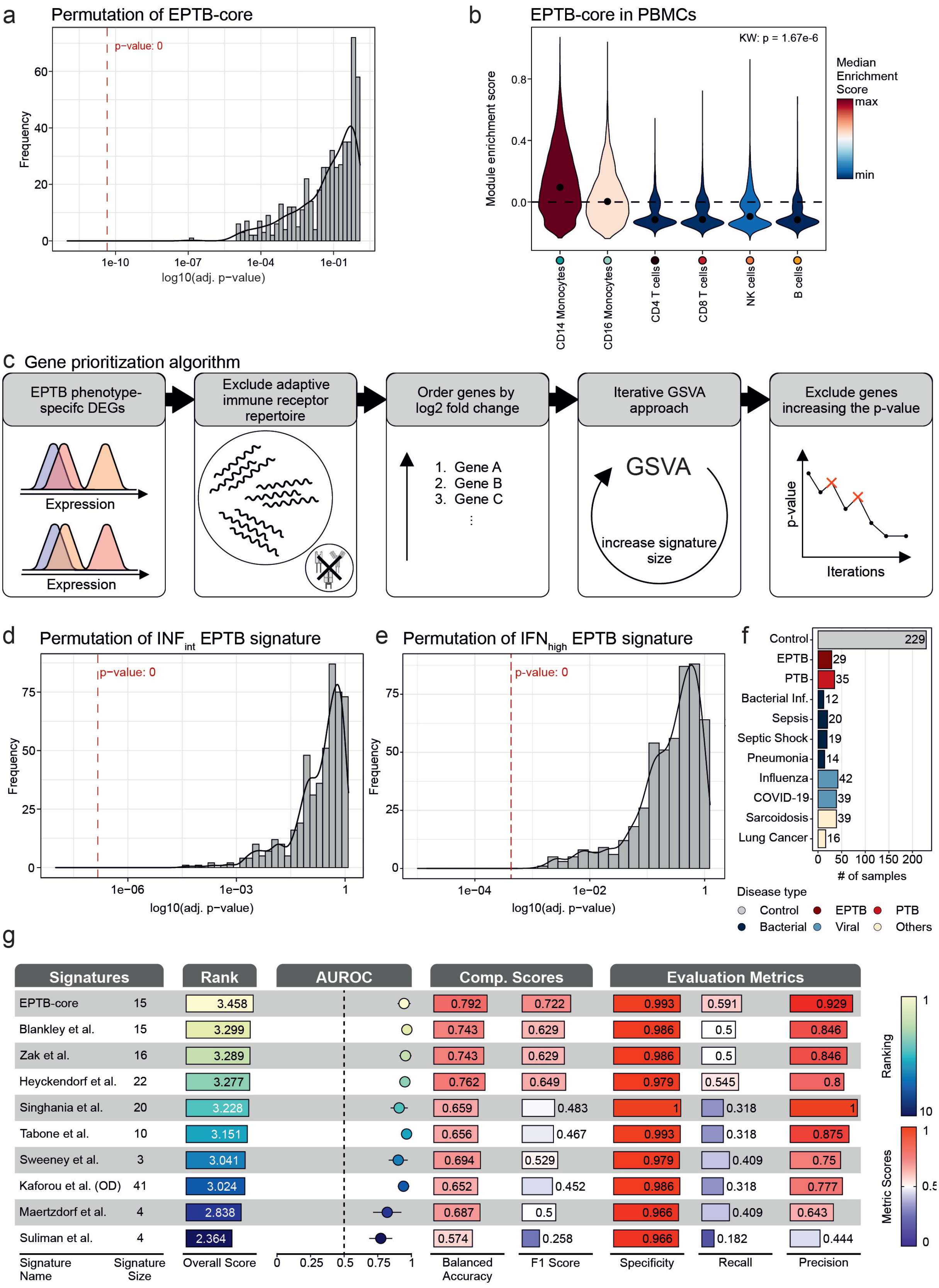
a
Permutation of EPTB-core
p-value: 0
Frequency
log10(adj. p-value)
b
EPTB-core in PBMCs
KW: p = 1.67e-6
Module enrichment score
Median Enrichment Score
max
min
CD14 Monocytes
CD16 Monocytes
CD4 T cells
CD8 T cells
NK cells
B cells
c Gene prioritization algorithm
EPTB phenotype-specifc DEGs
Expression
Expression
Exclude adaptive immune receptor repertoire
Order genes by log2 fold change
1. Gene A
2. Gene B
3. Gene C
Iterative GSVA approach
GSVA
increase signature size
Exclude genes increasing the p-value
p-value
Iterations
d
Permutation of INF$_{int}$ EPTB signature
p-value: 0
Frequency
log10(adj. p-value)
e
Permutation of IFN$_{high}$ EPTB signature
p-value: 0
Frequency
log10(adj. p-value)
f
Control 229
EPTB 29
PTB 35
Bacterial Inf. 12
Sepsis 20
Septic Shock 19
Pneumonia 14
Influenza 42
COVID-19 39
Sarcoidosis 39
Lung Cancer 16
# of samples
Disease type
Control
EPTB
PTB
Bacterial
Viral
Others
g
Signatures
Rank
AUROC
Comp. Scores
Evaluation Metrics
EPTB-core 15 3.458 0.792 0.722 0.993 0.591 0.929
Blankley et al. 15 3.299 0.743 0.629 0.986 0.5 0.846
Zak et al. 16 3.289 0.743 0.629 0.986 0.5 0.846
Heyckendorf et al. 22 3.277 0.762 0.649 0.979 0.545 0.8
Singhania et al. 20 3.228 0.659 0.483 1 0.318 1
Tabone et al. 10 3.151 0.656 0.467 0.993 0.318 0.875
Sweeney et al. 3 3.041 0.694 0.529 0.979 0.409 0.75
Kaforou et al. (OD) 41 3.024 0.652 0.452 0.986 0.318 0.777
Maertzdorf et al. 4 2.838 0.687 0.5 0.966 0.409 0.643
Suliman et al. 4 2.364 0.574 0.258 0.966 0.182 0.444
Signature Name
Signature Size
Overall Score
Balanced Accuracy
F1 Score
Specificity
Recall
Precision
Ranking
Metric Scores

Supplementary Fig. 14: a) p-value distribution of GSVA enrichment results of 500 random, unique gene sets on a log10 scale. Gene set size was based on the size of the EPTB-core signature. Statistics were computed using an unpaired two-sided Wilcoxon test followed by a Benjamini-Hochberg adjustment. The red dashed line represents the adjusted p value from GSVA enrichment of the EPTB-core signature. **b)** Violin plot of module ES of the EPTB-core signature in all cell types with more than 2,000 cells. Statistics were computed with a Kruskal-Wallis test per donor. **c)** Schematic of the gene prioritization algorithm. Created in BioRender. Dahm, K. (2025) https://BioRender.com/qxdus20. **d,e)** p-value distribution of GSVA enrichment results of 500 random, unique gene sets on a log10 scale. Gene set size was based on the size of the INF_{int} EPTB signature and INF_{high} EPTB signature, respectively. Statistics were computed between INF_{int} EPTB patients or INF_{high} EPTB patients and EPTB immunotypes together with the healthy controls, respectively, using an unpaired two-sided Wilcoxon test followed by a Benjamini-Hochberg adjustment. The red dashed line represents the adjusted p value from GSVA enrichment of the INF_{int} EPTB signature and INF_{high} EPTB signature, respectively. **f)** Bar plot of the number of samples included in the 'Other disease' (OD) data set split by disease and colored by disease type. **g)** Classifier overview of the EPTB-core and 9 published pulmonary TB (PTB) signatures in identifying EPTB and PTB from other diseases and healthy controls. The first bar plot represents the overall score of the area under the receiver operating characteristic curve (AUROC), specificity, precision and recall coloured by ranking of the signature. Point range plot depicts the AUROC and the 95 % confidence interval of the respective AUROC. The following bar plots depict the balanced accuracy, specificity, precision, recall and F1 score of each signature. Source data are provided as a Source Data file for the respective figures.

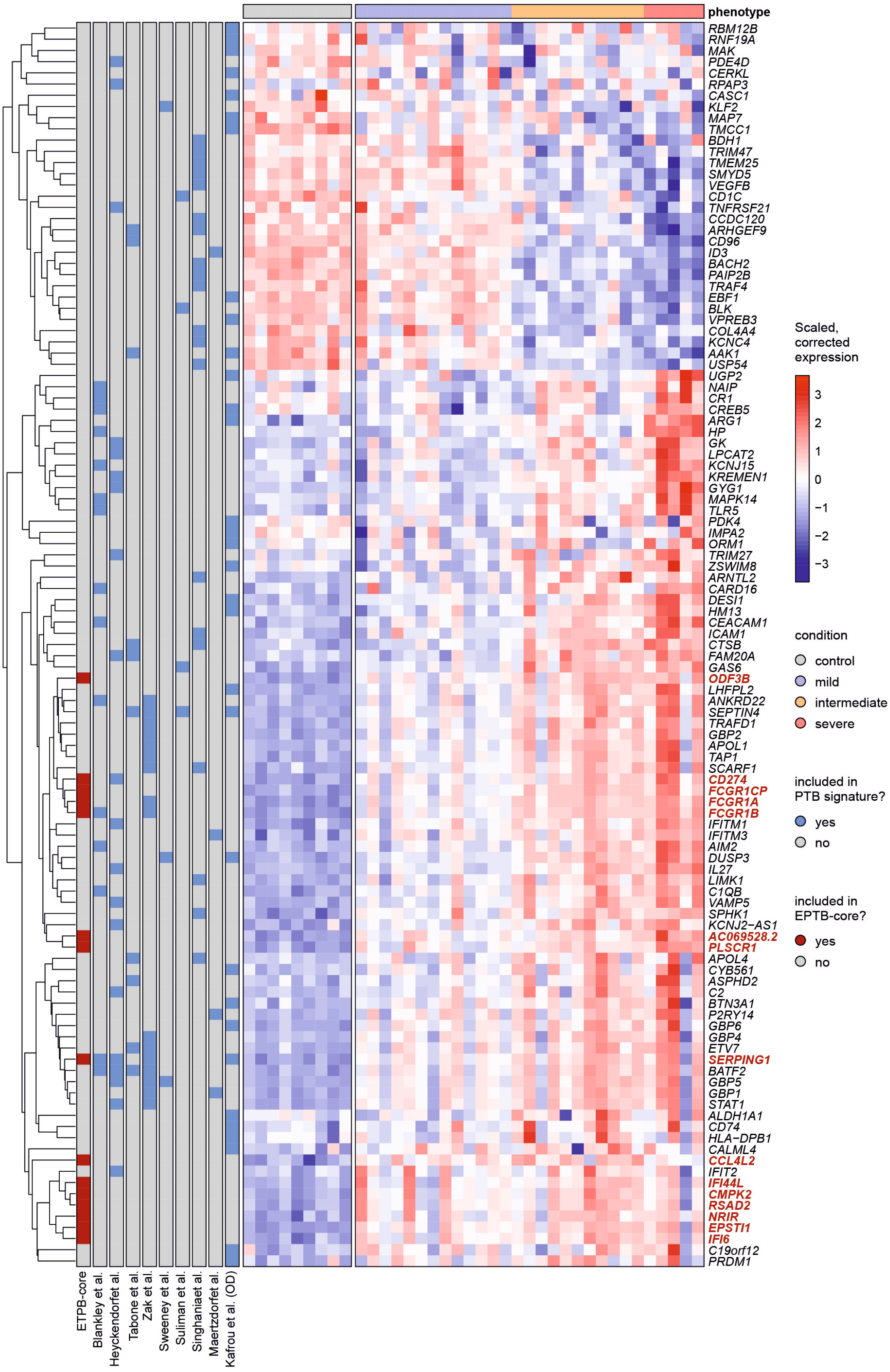

Supplementary Fig. 15: Heatmap of scaled, batch-corrected expression of EPTB-core and 9 published PTB signature. Presence of genes in the respective signatures is indicated and EPTB-core signature genes are highlighted.

Supplementary Table 1: Patient information

	Sex	Age at diagnosis	Country of Origin	Staging RNAseq	Extrapulmonary organ involvement (imaging based)	Extrapulmonary sample leading to diagnosis	Mtb complex isolate	Mtb strain resistance profile	Quantiferon-Gold result	HIV status
**EX-TB 001**	female	44	Ghana	**low**	isolated cervical lymph node	lymph node	M. tuberculosis	DS-TB	positive	neg.
**EX-TB 002**	female	24	Bulgaria	**low**	isolated cervical lymph node	lymph node	M. tuberculosis	DS-TB	positive	neg.
**EX-TB 004**	female	41	Morocco	**low**	isolated cervical lymph node	lymph node (histopathology)*	Unknown	n/a	positive	neg.
**EX-TB 007**	male	22	Germany	**low**	isolated cervical lymph node	lymph node	M. tuberculosis	DS-TB	positive	neg.
**EX-TB 013**	female	40	Germany	**low**	isolated cervical lymph node	lymph node	M. tuberculosis	DS-TB	positive	neg.
**EX-TB 017**	female	40	Germany	**low**	isolated cervical lymph node	lymph node	M. bovis	DS-TB	positive	neg.
**EX-TB 026**	female	44	Turkey	**low**	isolated cervical lymph node	lymph node (histopathology)**	Unknown	n/a	not available	neg.
**EX-TB 033**	male	30	India	**low**	isolated cervical lymph node	lymph node	M. tuberculosis	Isoniazid res.	positive	neg.
**EX-TB 038**	female	32	Nepal	**low**	isolated cervical lymph node	lymph node	M. tuberculosis	DS-TB	positive	neg.
**EX-TB 008**	male	18	Germany	**low**	mediastinal lymph nodes, pleural/pericardial effusions	Sputum culture positive - no lesions in computed tomography of lungs	M. tuberculosis	DS-TB	positive	neg.
**EX-TB 023**	male	32	Nigeria	**low**	abdominal lymph nodes	peritoneum	M. tuberculosis	DS-TB	positive	neg.
**EX-TB 025**	female	25	Somalia	**low**	isolated pleural tuberculosis	pleura	M. tuberculosis	DS-TB	positive	neg.
**EX-TB 032**	male	67	Germany	**low**	isolated joint (knee)	bone biopsy	M. tuberculosis	DS-TB	positive	neg.
**EX-TB 003**	female	70	Turkey	**intermediate**	isolated cervical lymph node	lymph node	M. bovis	DS-TB	positive	neg.
**EX-TB 031**	male	66	Afghanistan	**intermediate**	isolated cervical lymph node	lymph node	M. tuberculosis	DS-TB	positive	pos.
**EX-TB 019**	male	20	Somalia	**intermediate**	multiple lymph nodes (mediastinum), pleura	lymph node	M. tuberculosis	DS-TB	positive	neg.
**EX-TB 021**	female	25	Simbabwe	**intermediate**	multiple lymph nodes (cervical, mediastinal, infracranial, retroclaviculary, hepatic, gastric, inguinal, retroperitoneal, paraaortic, iliacal)	lymph node	M. tuberculosis	DS-TB	positive	neg.
**EX-TB 028**	female	44	Indonesia	**intermediate**	multiple lymph nodes (retroclavicular, mediastinal, paraaortic, iliacal on both sides, liver hilum)	lymph node	M. tuberculosis	DS-TB	positive	neg.
**EX-TB 030**	male	27	Tansania	**intermediate**	multiple lymph nodes (cervical both sides)	lymph node	M. tuberculosis	DS-TB	positive	neg.
**EX-TB 006**	male	38	Pakistan	**intermediate**	multiple lymph nodes (cervical, paratracheal), sternum	lymph node	M. tuberculosis	DS-TB	positive	neg.
**EX-TB 009**	male	28	Cameroon	**intermediate**	bone	paravertebral tumor	M. tuberculosis	Isoniazid res.	positive	neg.
**EX-TB 012**	male	25	Germany	**intermediate**	multiple lymph nodes (abdominal), soft tissue	abdominal lymph node	M. tuberculosis	DS-TB	positive	neg.
**EX-TB 014**	male	80	Turkey	**intermediate**	multiple lymph nodes, esophagus	lymph node	M. tuberculosis	Isoniazid res.	positive	neg.
**EX-TB 016**	male	53	Pakistan	**intermediate**	multiple lymph nodes, soft tissue, bones	bone abscess	M. tuberculosis	DS-TB	positive	neg.
**EX-TB 010**	male	25	Eritrea	**high**	bones (multiple lesions), abscesses	bone abscess/intraspinal abscess	M. tuberculosis	DS-TB	positive	neg.
**EX-TB 011**	female	32	Eritrea	**high**	peritoneum, lymph nodes (abdominal), liver, soft tissue, pleura	liver biopsy	M. tuberculosis	DS-TB	positive	neg.
**EX-TB 018**	male	50	Tansania	**high**	pleura, multiple lymph nodes	pleural biopsy	M. tuberculosis	DS-TB	positive	neg.
**EX-TB 022**	male	52	Turkey	**high**	testicles, colon, multiple lymph nodes	testicles (histopathology)***	Unknown	n/a	positive	neg.
**EX-TB 024**	male	22	Mauritius	**high**	peritoneum, pleura, multiple lymph nodes (mediastinal, abdomen), bones (spine), soft tissue	lymph node	M. bovis	DS-TB	positive	neg.

*Fibrotic lymph nodes with pronounced, often necrotizing and confluent epithelioid cell granulomatous inflammation.
**Confluent epithelioid cell granulomas containing giant cells, partly with central necrosis. Ziehl-Neelsen staining: mainly acid-fast rods within giant cells.
***Caseating granuloma.

Supplementary Table 2: Overview of signature genes

EPTB-core	INF_{int} EPTB signature	INF_{high} EPTB signature
CD274	*S100B*	*H2BC7*
IFI6	*CFD*	*CD177*
EPSTI1	*CCL3L3*	*ZDHHC19*
RSAD2		*HP*
CMPK2		*HTRA3*
IFI44L		*MMP8*
SERPING1		
FCGR1A		
ODF3B		
PLSCR1		
FCGR1B		
NRIR		
FCGR1CP		
CCL4L2		
AC069528.2		

Supplementary Table 3: List of all flow cytometry antibodies used in this study

Panel 1					Panel 2				
**Marker**	**Fluorophore**	**Dilution**	**Supplier**	**Reference Number**	**Marker**	**Fluorophore**	**Dilution**	**Supplier**	**Reference Number**
**CD45RA**	BUV395	1/100	BD Biosciences	740298	**CD14**	BUV395	1/100	BD Biosciences	563562
**IgM**	BUV496	1/100	BD Biosciences	750366	**CD1c**	BUV496	1/100	BD Biosciences	750182
**IgG**	BUV615	1/50	BD Biosciences	751180	**CD123**	BUV615	1/100	BD Biosciences	751315
**CD8**	BUV737	1/100	BD Biosciences	612755	**HLA-DR**	BUV737	1/100	BD Biosciences	752496
**CD24**	BUV805	1/100	BD Biosciences	742010	**CD16**	BUV805	1/100	BD Biosciences	748850
**TIM-3**	BV421	1/50	Biolegend	345007	**CD86**	BV421	1/100	Biolegend	305425
**CD4**	BV510	1/100	Biolegend	344633	**CD11b**	BV510	1/100	Biolegend	301333
**CD19**	BV570	1/25	Biolegend	302235	**CD19**	BV570	1/50	Biolegend	302235
**CD138**	BV605	1/50	Biolegend	356519	**CD127**	BV605	1/100	Biolegend	351333
**CD25**	BV650	1/100	Biolegend	302633	**CD56**	BV650	1/100	Biolegend	318343
**CD38**	BV711	1/50	Biolegend	303527	**CD11c**	BV711	1/100	Biolegend	301629
**CD127**	BV785	1/50	Biolegend	351329	**CD163**	BV785	1/50	Biolegend	333631
**CD62L**	FITC	1/100	Biolegend	304803	**BDCA-2 (CD303)**	FITC	1/50	Biolegend	354207
**IgA**	PerCP/Cy5.5	1/50	Miltenyi	130-114-004	**CD94**	PerCP/Cy5.5	1/50	Biolegend	305514
**TIGIT**	PE	1/50	Biolegend	372703	**CD80**	PE	1/100	Biolegend	305207
**CXCR5**	PE/Dazzle	1/50	Biolegend	356927	**NKG2D**	PE/Dazzle	1/50	Biolegend	320827
**CD27**	PE/Cy7	1/50	Biolegend	356411	**PD-L1 (MIH3)**	PE/Cy7	1/100	Biolegend	374505
**PD1**	APC/AF647	1/100	Biolegend	379207	**NKp44**	APC/AF647	1/50	Biolegend	325109
**CD3**	Alexa700	1/100	Biolegend	344821	**CD3**	Alexa700	1/100	Biolegend	344821
**Zombie NIR fixable viability dye**	APC/Cy7	1/200	Biolegend	423105	**Zombie NIR fixable viability dye**	APC/Cy7	1/200	Biolegend	423105

References

1 Schulte-Schrepping, J. *et al.* Severe COVID-19 Is Marked by a Dysregulated Myeloid Cell Compartment. *Cell* **182**, 1419-1440 e1423 (2020). https://doi.org:10.1016/j.cell.2020.08.001

2 Hillman, H. *et al.* Single-cell profiling reveals distinct subsets of CD14+ monocytes drive blood immune signatures of active tuberculosis. *Front Immunol* **13**, 1087010 (2022). https://doi.org:10.3389/fimmu.2022.1087010

3 Yanagihara, A. *et al.* A Th1-like CD4(+) T-cell Cluster That Predicts Disease-free Survival in Early-stage Lung Cancer. *Cancer Res Commun* **3**, 1277-1285 (2023). https://doi.org:10.1158/2767-9764.CRC-23-0167

4 Zhang, Y. *et al.* A single-cell atlas of the peripheral immune response in patients with influenza A virus infection. *iScience* **26**, 108507 (2023). https://doi.org:10.1016/j.isci.2023.108507
