# Supplementary material for: Deep immune profiling delineates hallmarks of disease heterogeneity in extrapulmonary tuberculosis
Source: Nat Commun. 2025 Nov 10;16:9662. doi: 10.1038/s41467-025-65561-x (PMC12603278; doi:10.1038/s41467-025-65561-x)
Supplement: Supplementary file 2 — Description of Additional Supplementary Files [file 41467_2025_65561_MOESM2_ESM.pdf]

Title: Supplementary Data 1

Description: Functional enrichment of DEGs related to Figure 1.See uploaded excel file.

Title: Supplementary Data 2

Description: List of AbSeq markers for scRNA-seq. See uploaded excel file.

Title: Supplementary Data 3:

Description: Functional enrichment of DEGs related to Figure 4. See uploaded excel file.
