# Supplementary material for: Deep immune profiling delineates hallmarks of disease heterogeneity in extrapulmonary tuberculosis
Source: Nat Commun. 2025 Nov 10;16:9662. doi: 10.1038/s41467-025-65561-x (PMC12603278; doi:10.1038/s41467-025-65561-x)
Supplement: Supplementary file 6 — Reporting Summary [file 41467_2025_65561_MOESM6_ESM.pdf]

nature portfolio

Corresponding author(s): Jan Rybniker, Thomas Ulas

Last updated by author(s): Oct 1, 2025

Reporting Summary

Nature Portfolio wishes to improve the reproducibility of the work that we publish. This form provides structure for consistency and transparency in reporting. For further information on Nature Portfolio policies, see our Editorial Policies and the Editorial Policy Checklist.

Statistics

For all statistical analyses, confirm that the following items are present in the figure legend, table legend, main text, or Methods section.

n/a	Confirmed	
[ ]	[x]	The exact sample size (n) for each experimental group/condition, given as a discrete number and unit of measurement
[ ]	[x]	A statement on whether measurements were taken from distinct samples or whether the same sample was measured repeatedly
[ ]	[x]	The statistical test(s) used AND whether they are one- or two-sided<br>*Only common tests should be described solely by name; describe more complex techniques in the Methods section.*
[ ]	[x]	A description of all covariates tested
[ ]	[x]	A description of any assumptions or corrections, such as tests of normality and adjustment for multiple comparisons
[ ]	[x]	A full description of the statistical parameters including central tendency (e.g. means) or other basic estimates (e.g. regression coefficient) AND variation (e.g. standard deviation) or associated estimates of uncertainty (e.g. confidence intervals)
[ ]	[x]	For null hypothesis testing, the test statistic (e.g. F, t, r) with confidence intervals, effect sizes, degrees of freedom and P value noted<br>*Give P values as exact values whenever suitable.*
[x]	[ ]	For Bayesian analysis, information on the choice of priors and Markov chain Monte Carlo settings
[ ]	[x]	For hierarchical and complex designs, identification of the appropriate level for tests and full reporting of outcomes
[ ]	[x]	Estimates of effect sizes (e.g. Cohen's d, Pearson's r), indicating how they were calculated

Our web collection on statistics for biologists contains articles on many of the points above.

Software and code

Policy information about availability of computer code

Data collection	NovaSeq6000 (Illumina), xPONENT software (Thermo Fisher Scientific)
Data analysis	bulk RNA-seq:<br>fastQC (v.0.11.9), multiQC (v1.14), STAR (v2.7.10b), Snakemake (v7.20.0), R (v4.1.0), R Studio (v1.4.1717), DESeq2 (v1.32.0), limma (v3.48.3), IHW (v1.20.0), apeglm (v1.14.0), clusterProfiler (v4.4.4), hcocena (v1.0.0), CIBERSORTx (https://cibersortx.stanford.edu/), caret (v7.0-1), randomForest (v4.7-1.2), MLeval (v0.3), plotROC (v2.3.1), pROC (v1.18.8), GSVA (v1.40.1), sva (3.40.0), rstatix (v0.7.0)<br><br>scRNA-seq:<br>bcl2fastq2 (v2.20), Cutadapt (v1.16), STAR (v2.6.1b), Dropseq-tools (v2.0.0), R (v4.1.0), R Studio (v1.4.1717), Seurat (v4.0.4), Vireo (v.0.5.6), cellsnp-lite (v1.2.2), scater (v1.20.1), harmony (v0.1.0), clusterProfiler (v4.0.5), rstatix (v0.7.0)<br><br>PROGENy analysis:<br>PROGENy (v1.22.0), decoupleR (v.2.6.0), R (v4.3.0), R Studio (v2023.3.0.386)<br><br>All original code has been deposited at GitLab under https://gitlab.dzne.de/ag-ulas/EPTB-RNAseq-Analysis and Zenodo under https://doi.org/10.5281/zenodo.17240509.<br><br>Cytokine array analysis:<br>GraphPad Prism 9.5.1

Flow cytometry:
FlowJo (v10.8.1), FlowSOM (v4.0.0)

For manuscripts utilizing custom algorithms or software that are central to the research but not yet described in published literature, software must be made available to editors and reviewers. We strongly encourage code deposition in a community repository (e.g. GitHub). See the Nature Portfolio guidelines for submitting code & software for further information.

Data

Policy information about availability of data

All manuscripts must include a data availability statement. This statement should provide the following information, where applicable:

- Accession codes, unique identifiers, or web links for publicly available datasets
- A description of any restrictions on data availability
- For clinical datasets or third party data, please ensure that the statement adheres to our policy

For the integration of scRNA-seq datasets, the following additional publically available datasets were used:
EGAS00001004571 [https://ega-archive.org/studies/EGAS00001004571], GSE243629 [https://www.ncbi.nlm.nih.gov/geo/query/acc.cgi?acc=GSE243629], and GSE215219 [https://www.ncbi.nlm.nih.gov/geo/query/acc.cgi?acc=GSE215219].
For the integration of 'Other Disease' datasets, the following additional publically available datasets were used:
GSE42834 [https://www.ncbi.nlm.nih.gov/geo/query/acc.cgi?acc=GSE42834], GSE68310 [https://www.ncbi.nlm.nih.gov/geo/query/acc.cgi?acc=GSE68310], EGAS00001004503 [https://ega-archive.org/studies/EGAS00001004503], and GSE154918 [https://www.ncbi.nlm.nih.gov/geo/query/acc.cgi?acc=GSE154918].
The whole blood RNA-seq and scRNA-seq data generated in this study have been deposited at the European Genome-phenome Archive (EGA) under accession codes EGAS50000000668 [https://ega-archive.org/studies/EGAS50000000668] and EGAS50000000758 [https://ega-archive.org/studies/EGAS50000000758], respectively, which is hosted by the EBI and the CRG. Restrictions apply regarding the availability of patient-derived biosamples. Source data are provided with this paper.
Requests for access to biological material should be directed to the corresponding authors. Requests are subject to ethical review and approval by the Ethics Committee of the University Hospital Cologne, and certain restrictions may apply

Research involving human participants, their data, or biological material

Policy information about studies with human participants or human data. See also policy information about sex, gender (identity/presentation), and sexual orientation and race, ethnicity and racism.

Reporting on sex and gender	The sex of study participants (based on self report) is provided in Supplementary Table 1. No participants were excluded based on their sex. No sex analysis was conducted, because of the limited amount of study participants.
Reporting on race, ethnicity, or other socially relevant groupings	Not applicable
Population characteristics	This is provided in supplementary table 1
Recruitment	Patients were recruited under the umbrella of the Cologne EX-TB study.
Ethics oversight	Ethics committee of the University Hospital Cologne (identifier: 18-079)

Note that full information on the approval of the study protocol must also be provided in the manuscript.

Field-specific reporting

Please select the one below that is the best fit for your research. If you are not sure, read the appropriate sections before making your selection.

☒ Life sciences ☐ Behavioural & social sciences ☐ Ecological, evolutionary & environmental sciences

For a reference copy of the document with all sections, see nature.com/documents/nr-reporting-summary-flat.pdf

Life sciences study design

All studies must disclose on these points even when the disclosure is negative.

Sample size	Sample size was dictated by the number of available patients and biosamples at the end of recruitment
Data exclusions	There was no data exlusion
Replication	Patient derived biosamples were used for this study. Technical replicates were applied. All attempts at replication were successful.
Randomization	Not applicable. Observational study. Healthy control biosamples were used as controls for the study
Blinding	The investigators performing the flow cytometry and Luminex experiments were also blinded, as alternative sample names were used. The Bioinformatitians evaluating bulk RNAseq were also blinded, as they were not aware of the grouping. For single cell RNAseq, individual patients had to be chosen for analysis. To ensure proper representation of the groups, blinding was not possible here.

Reporting for specific materials, systems and methods

We require information from authors about some types of materials, experimental systems and methods used in many studies. Here, indicate whether each material, system or method listed is relevant to your study. If you are not sure if a list item applies to your research, read the appropriate section before selecting a response.

Materials & experimental systems

n/a	Involved in the study
☐	☒ Antibodies
☒	☐ Eukaryotic cell lines
☒	☐ Palaeontology and archaeology
☒	☐ Animals and other organisms
☐	☒ Clinical data
☒	☐ Dual use research of concern
☒	☐ Plants

Methods

n/a	Involved in the study
☒	☐ ChIP-seq
☐	☒ Flow cytometry
☒	☐ MRI-based neuroimaging

Antibodies

Antibodies used	FACS: All FACS antibodies are listed in supplementary table 3; ScRNA-seq: BD Biosciences AbSeq Immune Discovery Panel, Cat#25970
Validation	The used antibodies were titrated by the investigators before use and validated for the use in flow cytometry on human samples by the manufacturers (refer to manufacturer websites, manufacturers listed in supplementary table 3)

Clinical data

Policy information about clinical studies

All manuscripts should comply with the ICMJE guidelines for publication of clinical research and a completed CONSORT checklist must be included with all submissions.

Clinical trial registration	NCT06875336
Study protocol	Study protocol was written in German, can be provided upon request.
Data collection	Patients were enroled from August 2018 and September 2021 at the University Hospital Cologne, Devision of Infectious Diseases
Outcomes	Observational study, not applicable

Plants

Seed stocks	*Report on the source of all seed stocks or other plant material used. If applicable, state the seed stock centre and catalogue number. If plant specimens were collected from the field, describe the collection location, date and sampling procedures.*
Novel plant genotypes	*Describe the methods by which all novel plant genotypes were produced. This includes those generated by transgenic approaches, gene editing, chemical/radiation-based mutagenesis and hybridization. For transgenic lines, describe the transformation method, the number of independent lines analyzed and the generation upon which experiments were performed. For gene-edited lines, describe the editor used, the endogenous sequence targeted for editing, the targeting guide RNA sequence (if applicable) and how the editor was applied.*
Authentication	*Describe any authentication procedures for each seed stock used or novel genotype generated. Describe any experiments used to assess the effect of a mutation and, where applicable, how potential secondary effects (e.g. second site T-DNA insertions, mosiacism, off-target gene editing) were examined.*

Flow Cytometry

Plots

Confirm that:

- ☒ The axis labels state the marker and fluorochrome used (e.g. CD4-FITC).
- ☒ The axis scales are clearly visible. Include numbers along axes only for bottom left plot of group (a 'group' is an analysis of identical markers).
- ☒ All plots are contour plots with outliers or pseudocolor plots.
- ☒ A numerical value for number of cells or percentage (with statistics) is provided.

Methodology

Sample preparation	Peripheral blood mononuclear cells obtained from patients and controls were stained according to the methods section of the manuscript
Instrument	BD FACSymphony A3
Software	Data was collected using the BD FACSDiva software and analyzed using FlowJo (Version 10.8.1). Statististical analysis was performed using Graphpad Prism 9.5.1 software.
Cell population abundance	Only viable, single cells were included in the analysis, ensuring high purity of the samples. While some rare cell types were only present in low abundance, most cell-populations discussed in this manuscript were present in reasonably high frequencies, which allowed accurate gating.
Gating strategy	Only viable cells were used for the analysis, which were identified by using a viability dye. Debris and doublet cells were excluded from all analyses by using a FCS-A/FCS-H gate followed by a FCS/SSC gate as indicated in the gating strategy provided in the supplementary information (Supplementary Fig. 4&5). "positive" and "negative" staining cells were distinguished by gating clearly visible separate populations, which was kept consistent between donors where feasible.

☒ Tick this box to confirm that a figure exemplifying the gating strategy is provided in the Supplementary Information.
